# A primary cilia–autophagy axis in hippocampal neurons is essential to maintain cognitive resilience

**DOI:** 10.1038/s43587-024-00791-0

**Published:** 2025-02-21

**Authors:** Manon Rivagorda, David Romeo-Guitart, Victoria Blanchet, François Mailliet, Valérie Boitez, Natalie Barry, Dimitrije Milunov, Eleni Siopi, Nicolas Goudin, Stéphanie Moriceau, Chiara Guerrera, Michel Leibovici, Soham Saha, Patrice Codogno, Eugenia Morselli, Etienne Morel, Anne-Sophie Armand, Franck Oury

**Affiliations:** 1 Université Paris Cité, INSERM UMR-U1151, CNRS UMR-8253, Institut Necker Enfants Malades, Team 8, Paris, France; 2 MedInsights SAS, Paris, France; 3 Platform for Image Analyses, Structure Fédérative de Recherche Necker, INSERM US24/CNRS UAR 3633, Paris, France; 4 Platform for Neurobehavioral and Metaboblism, Institut Imagine, Structure Fédérative de Recherche Necker, 26 INSERM US24/CNRS UAR, Paris, France; 5 Platform for Proteomic Analyses, Structure Fédérative de Recherche Necker, INSERM US24/CNRS UAR 3633, Paris, France; 6 Université Paris Cité, INSERM UMR-U1151, CNRS UMR-8253, Institut Necker Enfants Malades, Team 6, Paris, France; 7 Department of Basic Sciences, Faculty of Medicine and Sciences, Universidad San Sebastián, Santiago, Chile; 8 Present Address: Institute for Experimental and Clinical Pharmacology and Toxicology, Center of Brain, Behavior and Metabolism, University of Lübeck, Lübeck, Germany

**Keywords:** Macroautophagy, Neurophysiology, Ageing

## Abstract

Blood-borne factors are essential to maintain neuronal synaptic plasticity and cognitive resilience throughout life. One such factor is osteocalcin (OCN), a hormone produced by osteoblasts that influences multiple physiological processes, including hippocampal neuronal homeostasis. However, the mechanism through which this blood-borne factor communicates with neurons remains unclear. Here we show the importance of a core primary cilium (PC) protein–autophagy axis in mediating the effects of OCN. We found that the OCN receptor GPR158 is present at the PC of hippocampal neurons and mediates the regulation of autophagy machinery by OCN. During aging, autophagy and PC core proteins are reduced in neurons, and restoring their levels is sufficient to improve cognitive impairments in aged mice. Mechanistically, the induction of this axis by OCN is dependent on the PC-dependent cAMP response element-binding protein signaling pathway. Altogether, this study demonstrates that the PC–autophagy axis is a gateway to mediate communication between blood-borne factors and neurons, and it advances understanding of the mechanisms involved in age-related cognitive decline.

## Abstract (web summary)

Exploring the molecular mechanisms by which the blood-borne factor osteocalcin promotes cognitive resilience, Rivagorda, Romeo-Guitart et al. identify a primary cilia axis through which osteocalcin promotes autophagy in neurons.

## Main

During aging, organisms undergo a decline in tissue function, leading to reduced systemic, cellular and organ performance, with several functional consequences, such as decreased cognitive capacity^1–3^. Among the different brain regions affected by aging, the hippocampus is particularly susceptible^3–5^. Central hallmarks of hippocampal aging include alterations in neuronal homeostasis and adaptive functional properties, leading to deficits in synaptic plasticity and memory^4,6–9^. Many efforts are now focused on the identification of molecular pathways involved in these processes to develop effective preventive or therapeutic strategies to treat age-related cognitive deficits.

Growing evidence has shown that administration of plasma derived from young animals is sufficient to improve hippocampal-dependent cognitive impairments in aged mice, underlying the importance of blood-circulating factors in brain aging^9–16^. Leveraging technological advances in omics analyses led to the identification of blood-borne factors that can foster neuronal homeostasis and cognitive resilience. Among them, we and others have identified the bone-derived hormone osteocalcin (OCN), which crosses the blood–brain barrier and impacts neuronal homeostasis and cognitive fitness via activation of its receptor GPR158, a G-protein-coupled receptor (GPCR) expressed in hippocampal neurons^17–19^. Circulating OCN levels are reduced in aged mice, whereas their restoration is sufficient to improve age-related cognitive deficits by the induction of autophagy machinery^18–20^.

Autophagy is a catabolic cellular process, whereby proteins and organelles are engulfed in autophagosomes (APs), formed by the activity of different autophagy proteins, and then transported to lysosomes for degradation, contributing directly to cell metabolism and energy production^21–23^. In terminally differentiated cells such as neurons, autophagy-mediated degradation of intracellular material is key to maintain cellular homeostasis and promotes cell survival^24–26^. However, beyond its basal functions, autophagy machinery could also be induced by various stimuli (stress, hormones and nutrients), which mediate the adaptive cellular responses to changes in environmental conditions^27–29^.

In the brain, stimulated autophagy promotes activity-dependent synaptic plasticity in hippocampal neurons and memory formation^20,30–33^. During aging, autophagy levels are reduced in the hippocampus, leading to a decline in the capacity of neurons to conserve their ability to integrate novel stimuli, and, thereby, in memory functions^20,33,34^. Importantly, administration of young blood is sufficient to restore the level of essential autophagy-related proteins in hippocampal neurons of old mice, which is a prerequisite to foster cognitive resilience^20^. Among these cognitive resilience factors, we and others identified OCN or growth differentiation factor 11 (GDF11)^20,34,35^. However, the regulatory mechanisms mediating the induction of neuronal autophagy by these blood-borne factors remain largely elusive.

Recent work in the kidney has shown that autophagic machinery can be mobilized in response to systemic stimuli by direct or indirect connection with core primary cilia (PC) proteins, namely intraflagellar transports (IFTs) (IFT20 and IFT88) or kinesin family member 3A (KIF3A)^36–38^. In neurons, the link between PC and autophagy machinery has not yet been characterized.

PC are non-motile single organelles, acting as cellular ‘antenna’ sensing and transmitting changes in the systemic milieu^39,40^. Importantly, mutations in ciliary proteins are implicated in a wide variety of genetic disorders, known as ciliopathies, which, among other phenotypes, lead to neurological defects^41^. Previous research indicates that depletion of core cilia proteins in different brain regions results in altered cognitive function^42–44^. Numerous studies also indicate that PC are hubs for various GPCRs, the largest families of membrane protein sensors, involved in many physiological processes, including hormonal systems^45–49^. Knowing that GPR158 is the receptor of OCN in hippocampal neurons led us to hypothesize that PC and/or core PC proteins constitute a gateway through which the blood factor OCN modulates neuronal autophagy machinery and, therefore, cognitive fitness.

In the present study, we confirmed that GPR158 could be localized at the PC and is required for OCN-dependent autophagy induction in hippocampal neurons. Consistently, we show here that core PC proteins are required for the mobilization of key autophagy players by OCN in hippocampal neurons. In aged hippocampi, neuronal PC present an abnormal morphology, correlated with a reduction of the major core ciliary protein levels. Conversely, restoration of their levels in old hippocampi is sufficient to improve age-related neuronal autophagy and cognitive deficits. Lastly, we show that core PC proteins are required to integrate the rejuvenating effects of OCN in the hippocampus, thereby ameliorating age-related cognitive deficits. Altogether, these findings identify a paradigm for the neuron/systemic milieu communication and advance understanding of the regulatory mechanism mediating the rejuvenating effects of the blood-borne factors, such as OCN, in hippocampal neurons.

## Results

### OCN triggers a modulation in PC-related protein expression

**Fig. 1:**
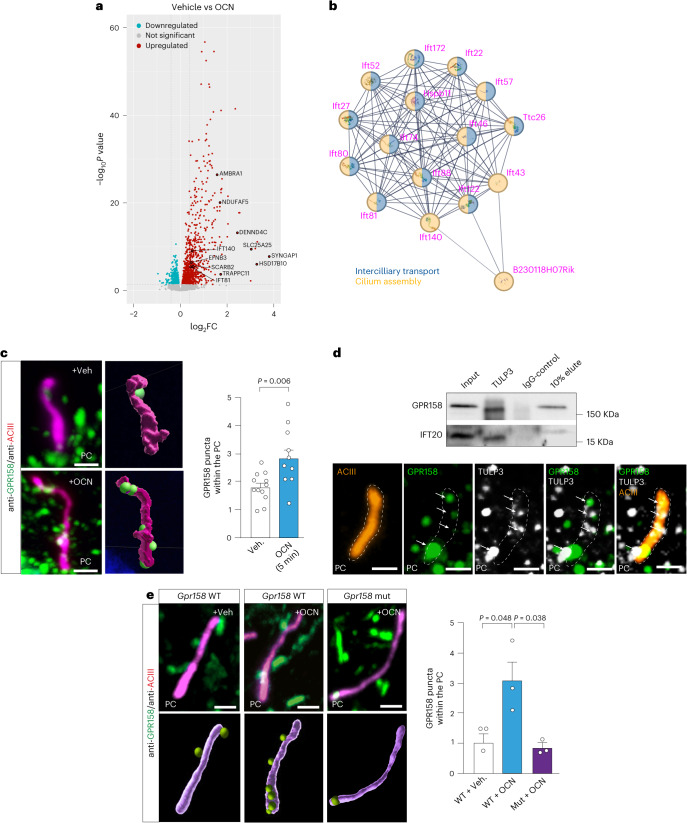
GPR158 localized in PC of hippocampal neurons. **a**, Volcano plot showing protein fold change between vehicle-treated and OCN-treated hippocampi. **b**, Protein ontology network showing enrichment for PC core proteins in the hippocampus treated with OCN versus vehicle. **c**, 2D image (left) and 3D rendering (right) of GPR158 (green) localization in PC from primary hippocampal neurons treated with vehicle or OCN. Scale bar, 1 µm. Graph bar represents the quantification of GPR158 spots in PC (vehicle *n* = 12, OCN *n* = 10, from two independent experiments). **d**, Co-immunoprecipitation of TULP3 with GPR158 or IFT20 in N2a cells and representative image of co-immunofluorescence of GPR158 (green) and TULP3 (white) in the PC stained with ACIII (orange) of hippocampal neurons. Scale bar, 1 µm. **e**, 2D image (top) and 3D rendering (bottom) of GPR158 (green) localization in PC from *Gpr158*^*−/−*^ primary hippocampal neurons infected with WT or mutated *Gpr158* and treated or not with OCN. Scale bar, 1 µm. Quantification of GPR158 spots found in each neuronal PC from three independent cultures (in 10–25 neurons per dot). PC was stained by ACIII in each corresponding panel. Data are represented as mean ± s.e.m. The *P* value was determined by two-tailed Student’s *t*-test compared to control group (**c**) or by one-way ANOVA followed by a post hoc Sidak correction (**e**). Source data

**Extended Data Fig. 1:**
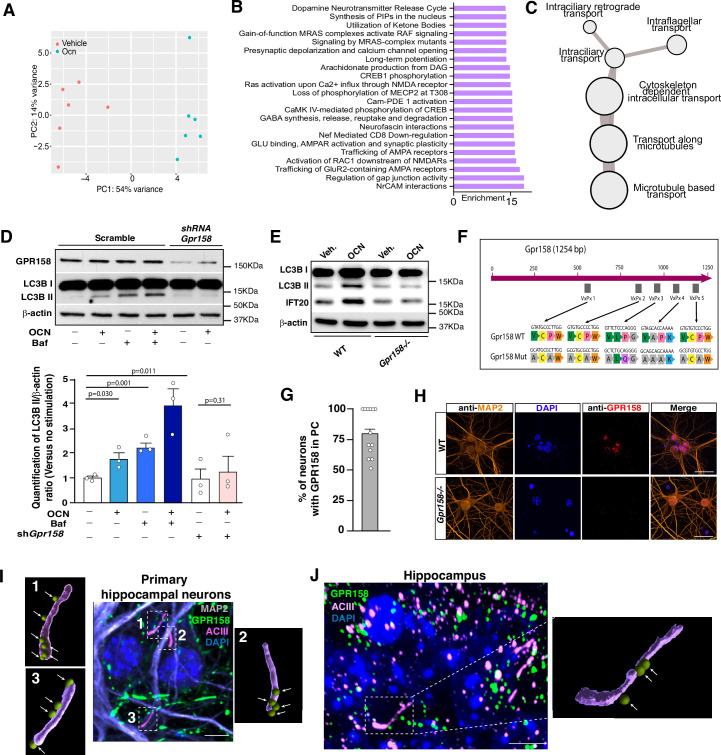
GPR158 is present at the PC of hippocampal neurons. **A**) PCA biplot showing that the proteomic datasets collected after stereotactic injections with either vehicle (NaCl) or OCN are orthogonal to each other. **B**) Bar graph representation of the top upregulated signaling pathways in differential analysis of hippocampi 4 h after stereotactic injections with either vehicle or OCN. **C**) Protein ontology network associated to PC-core proteins and PC functions enriched in the hippocampus after OCN treatment. **D**) Western blot analysis and quantification of LC3B-II accumulation in primary neurons infected with lentivirus expressing either shRNA-Gpr158 or shRNA-scramble. Independent neuronal cultures (n = 3) were treated with either vehicle, OCN, bafilomycin A1 (Baf), or OCN + Baf. **E**) Western blot analysis of LC3B-I, LC3B-II and IFT20 in WT and Gpr158-/- mouse hippocampi 4 h after hippocampal stereotactic injections with either vehicle or OCN. **F**) Schematic representation of the 5 discrete ciliary targeting (VXPX motif) sequences in the C-terminal tail of GPR158. We have generated a mutated Gpr158 version. **G**) Proportion of neuronal primary cilium presenting GPR158+ and ACIII+ puncta. **H**) Representative images of GPR158 staining in hippocampal cultures derived from either WT or Gpr158-/- mice. Scale bar=10μm. **I**-**J**) Representative images of co- immunofluorescence for GPR158 (green) and ACIII (orange) in hippocampal neurons from 3 independent experiments (**I**) and in WT hippocampal cross-section of the CA3 region of mice treated with OCN from 2 independent experiments (**J**). 1, 2, and 3 squares in I are 3D rendering images of GPR158 localization in PC from mature hippocampal neurons treated with vehicle or OCN. Scale bar=5μm. In all western blots, β-actin was used as a loading control for each sample. Data are expressed as mean ± SEM. The p values were determined by a one-tailed Student’s t-test. Source data

We previously showed that OCN can influence neuronal homeostasis and cognitive fitness by mobilizing autophagic machinery in hippocampal neurons^20^. Exploring the mechanism by which this regulation occurs, we performed comparative proteomic analysis in hippocampi from mice injected with vehicle or OCN. As a result, we found that OCN treatment in the hippocampus enhanced the levels of PC-related proteins and synaptic plasticity (Fig. 1a,b and Extended Data Fig. 1a–c). Interestingly, recent studies proposed that autophagic machinery can be triggered in response to various stimuli, such as serum deprivation and shear stress, by direct or indirect connection with core PC proteins, namely IFTs (such as IFT20 and IFT88) and kinesin (including KIF3A, a kinesin-2 family motor protein)^36–40^. These proteins allow the transportation of ciliary cargoes and are essential for the formation, maintenance and signaling activity of the PC^50,51^.

### GPR158 is present at the PC of hippocampal neurons

The PC was described as a signaling hub for various GPCRs that sense and transduce changes of the systemic milieu^46^. Interestingly, a key regulator of OCN’s influence on memory is GPR158, an orphan GPCR expressed in neurons of the cornu ammonis 3 (CA3) region of the hippocampus^18,19^. Using primary hippocampal neurons, we first confirmed that the enhancement of AP-like structure formation by OCN is dependent on GPR158 (Extended Data Fig. 1d), by measuring lipidation levels of the LC3B protein (LC3B-II) (a key readout in AP-like structure biogenesis) in the presence or absence of a lysosomal blocker (bafilomycin). This observation was further confirmed in vivo showing that local stereotactic OCN injections enhance hippocampal LC3B lipidation in wild-type (WT) but not in *Gpr158*^−/−^ mice (Extended Data Fig. 1E).

Remarkably, we identified five ciliary-targeting VxPx motifs at the C-terminal domain of GPR158, suggesting its possible localization in the PC (Extended Data Fig. 1f). Co-staining of endogenous GPR158 with neuronal ciliary protein adenylate cyclase 3 (ACIII) indicates its presence, at least partially, at the PC in hippocampal neurons, in cultures (in approximatively 75% of primary hippocampal neurons) and in mouse hippocampi (Fig. 1c and Extended Data Fig. 1g–j). Furthermore, we found that OCN treatment increases the number of GPR158-positive puncta associated with ACIII-positive ciliary plasma membrane (Fig. 1c). Endogenous GPR158 was further co-immunoprecipitated and co-localized with the ciliary protein Tubby-like protein 3 (TULP3), which is involved in the localization and trafficking of GPCRs to PC^52^ (Fig. 1d). By contrast, mutagenesis of the five ciliary-targeting VxPx motifs at the C-terminal domain of GPR158 reduced the presence of GPR158-positive puncta in neuronal PC structure (Fig. 1e). Altogether, these data indicate that the induction of autophagy by OCN in hippocampal neurons is dependent on GPR158 and that OCN can enhance the localization of this receptor to PC.

### Core PC proteins are required for OCN to induce autophagy

**Fig. 2:**
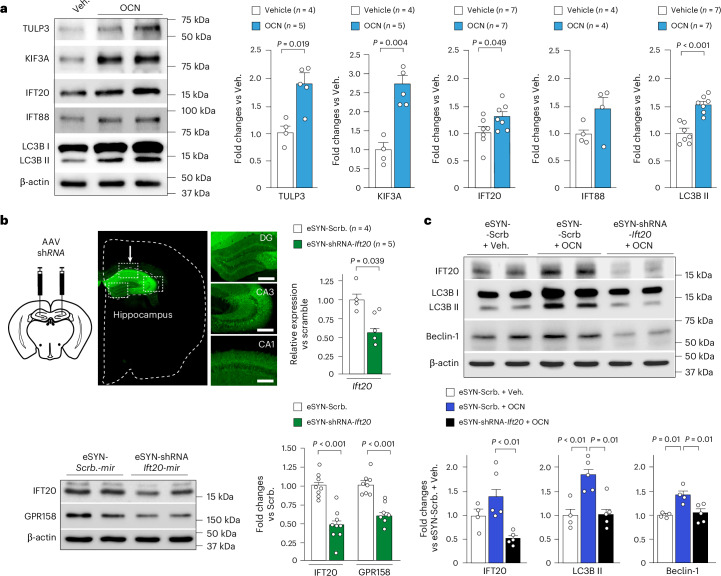
OCN–GPR158 coupling system requires core PC proteins to induce autophagy machinery in hippocampal neurons. **a**, Western blot and quantification analysis of TULP3, KIF3A, IFT20, IFT88 and LCB-I/II protein levels. **b**, Top panels are representative fluorescent images of brain cross-sections 3 weeks after injections with AAV-eSYN-shRNAmir-scramble expressing eGFP. Left panel is a panoramic view; right panels are focus views on the DG, CA3 and cornu ammonis 1 (CA1) regions, respectively. Scale bar, 100 µm. Top right panel is the relative expression of *Ift20* mRNA level in the dorsal hippocampus of mice 3 weeks after injection with AAV-eSYN-shRNA-scramble-mir or AAV-eSYN-shRNAmir*-Ift20*. Bottom panels represent western blot 3 weeks after hippocampal injections with AAV-eSYN-shRNA-scramble-mir or AAV-eSYN-shRNAmir*-Ift20* and respective quantification of IFT20 (eSYN-Scrb *n* = 8 and eSYN-shRNA-Ift20 *n* = 9) and GPR158 (eSYN-Scrb *n* = 8 and eSYN-shRNA-Ift20 *n* = 7). **c**, Western blot and quantification of IFT20 (eSYN-Scrb+veh *n* = 4, eSYN-Scrb+OCN *n* = 5, eSYN-shRNA-Ift20+OCN *n* = 5), LC3B-I/II (eSYN-Scrb+veh *n* = 4, eSYN-Scrb+OCN *n* = 5, eSYN-shRNA-Ift20+OCN, *n* = 5) and Beclin-1 (eSYN-Scrb+veh *n* = 4, eSYN-Scrb+OCN *n* = 4, eSYN-shRNA-Ift20+OCN, *n* = 5) proteins in hippocampi, 3 weeks after injections with AAV-*eSYN*-shRNA-*scramble-mir* or AAV-*eSYN*-shRNA-*Ift20-mir* and upon stereotactic injection of vehicle or OCN. Quantification is relative to injection with AAV-eSYN1-shRNA-scramble-mir treated with vehicle. β-actin was used as a loading control for each sample in all western blot analyses. All data are expressed as mean ± s.e.m., and *P* values were determined by one-tailed (**b**, top) or two-tailed (**a** and **b**, bottom) Student’s *t*-tests compared to vehicle or eSYN-Scbr. In **c**, the *P* values were determined by one-way ANOVA followed by Tukey’s multiple comparison test. Source data

**Extended Data Fig. 2:**
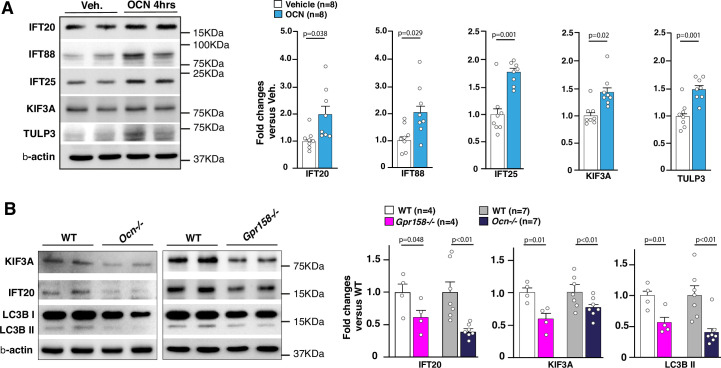
OCN enhances the level of core PC-proteins. **A**) Representative Western blot and quantification of IFT20, IFT88, IFT25, KIF3A and TULP3 in the hippocampus 4 h after local stereotactic injections of vehicle or OCN. **B**) Representative Western blot and quantification of KIF3A, IFT20 and LC3B-II accumulation in 3-month-old WT, *Ocn-/-* or *Gpr158-/-* mouse hippocampi. In all western blots, β-actin was used as a loading control for each sample. The p values were determined by a one-tailed (**B**) or two-tailed (**A**) Student’s t-test. Source data

**Extended Data Fig. 3:**
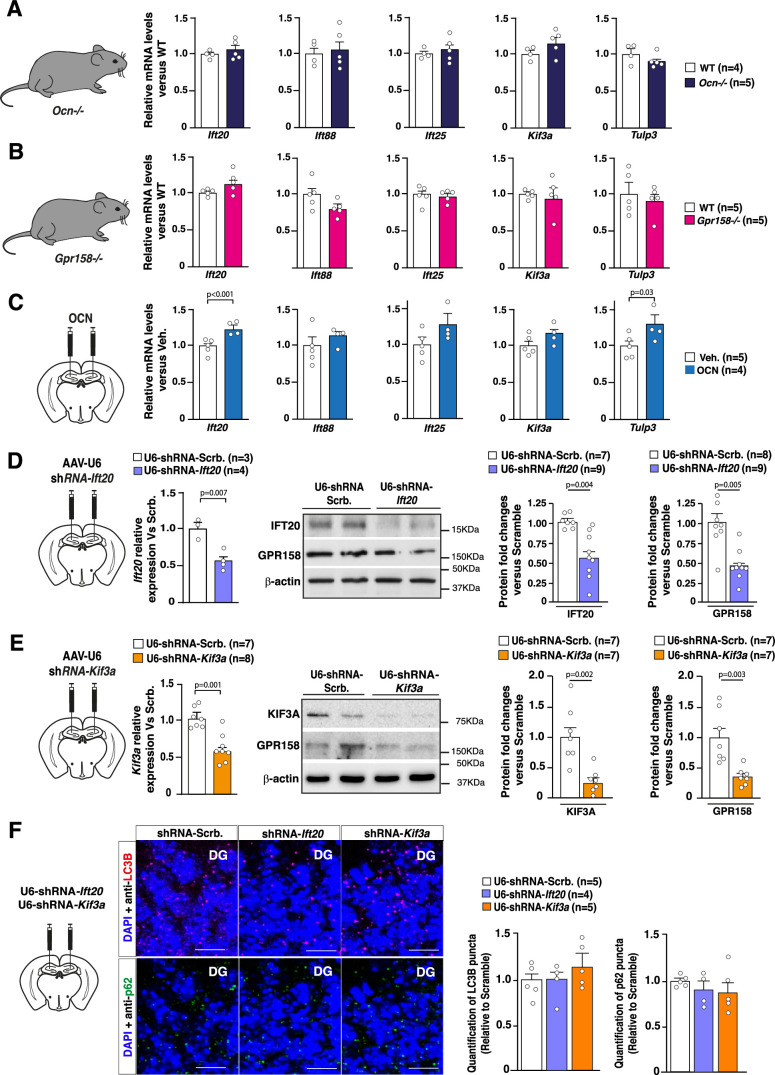
Reciprocal regulation of the GPR158-OCN coupling system and core PC protein levels. **A**-**C**) Relative gene expression of Ift20, Ift88, Ift25, Kif3a and Tulp3 in the hippocampus of either Ocn-/- (**A**), Gpr158-/- (**B**) or WT after stereotactic injections of OCN (**C**). The quantification is relative to WT (**A**-**B**) or WT after vehicle stereotactic injections (**C**). **D**-**E**) Relative gene expression (RT-qPCR) of either Ift20 or Kif3a and representative Western blots of IFT20, KIF3A and GPR158 in 3-month-old mice hippocampi, 3 weeks after hippocampal stereotactic injections with either AAV-U6-shRNA-Ift20 (**D**), AAV-U6-shRNA-Kif3a (**E**) or their respective controls. β-actin was used as a loading control for WB analyses. **F**) LC3B and SQSTM1/p62 immunofluorescence and punctae quantification performed on brain cross sections at the level of the hippocampal Dentate Gyrus (DG) performed in 3-month-old mice, 3 weeks after local stereotactic injections with either AAV-U6-shRNA-Ift20, AAV-U6-shRNA-Kif3a or AAV-U6-shRNA-Scramble. Scale bar: 7 μm. Data are expressed as mean ± SEM. The p-values were determined by a two-tailed Student’s t-test compared to control (vehicle or U6-shRNA-Scrb depending on the experiment). Source data

Next, we found that treatment of primary hippocampal neurons with OCN, or local stereotactic injections of OCN in mouse hippocampus, enhance the level of core PC proteins (TULP3, KIF3A, IFT20 and IFT88), together with lipidated LC3B (LC3B-II levels) (Fig. 2a and Extended Data Fig. 2a). By contrast, IFT20 and KIF3A protein levels are lower in *Ocn*^*−/−*^ and *Gpr158*^*−/−*^ mice than in WT littermates, which is correlated with a reduction of LC3B-II levels (Extended Data Fig. 2b). At the mRNA levels, we did not observe any significant changes of these core PC proteins in *Ocn*^*−/−*^ or *Gpr158*^*−/−*^ hippocampi, but we did observe a slight increase of *Ift20* and *Tulp3* transcript levels after OCN treatment (Extended Data Fig. 3a–c). Then, using stereotactic injections of adeno-associated virus (AAV) expressing small hairpin RNA (shRNA), we generated mouse models in which the intraflagellar *Ift20* (*AAV9-U6-shRNA-Ift20*) or the kinesin *Kif3a* (*AAV9-U6-shRNA-Kif3a*) proteins are selectively downregulated in the hippocampus. All in vivo experiments were performed on male mice in this study. These mice exhibit a significant reduction of hippocampal GPR158 levels in comparison to controls (*AAV-U6-shRNA-scramble* injected mice) (Extended Data Fig. 3d,e). Similar results were obtained after *Ift20* downregulation in mature hippocampal neurons, generated by local stereotactic injections of AAVs expressing shRNAmir for *Ift20* (AAV9-eSYN-*Ift20*-shRNAmir) under the control of the neuronal eSynapsin 1 (e-SYN) promoter (Fig. 2b). Moreover, we found that *Ift20* downregulation in mature hippocampal neurons is sufficient to abolish the induction of the autophagy machinery by OCN (Fig. 2c).

Altogether, these data demonstrate that core PC proteins are required to mediate the induction of autophagy machinery in hippocampal neurons, by the coupling of OCN and GPR158.

### Core PC proteins modulate autophagy in hippocampal neurons

**Fig. 3:**
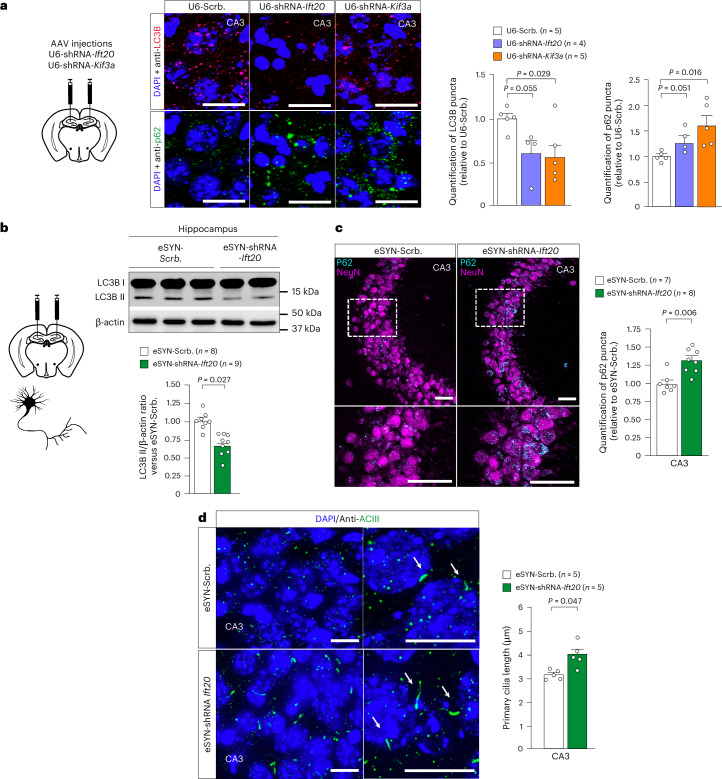
Downregulation of PC core proteins impairs autophagy machinery in hippocampal neurons. **a**, LC3B and SQSTM1/p62 immunofluorescence and puncta quantification performed on brain cross-sections at the level of the hippocampal CA3 region from 3-month-old mice, 3 weeks after local stereotactic injections with AAV-U6-shRNA-*Ift20*, AAV-U6-shRNA-*Kif3a* or AAV-U6-shRNA-scramble. Scale bar, 20 µm. **b**, Representative western blot image and quantification of LC3B-II accumulation in 3-month-old mouse hippocampi, 3 weeks after local stereotactic injections with either eSYN-shRNA-*Ift20mir* or eSYN-shRNA-scramble-mir (two independent cohorts). β-actin was used as a loading control for each sample. **c**, SQSTM1/p62 immunofluorescence (blue) and punctae quantification in the CA3 region of the hippocampi of 3-month-old mice, 3 weeks after local stereotactic injections of AAVs expressing either eSYN-shRNA-*Ift20-mir* or eSYN-shRNA-scramble-mir. NeuN staining (purple) was used to label neuronal nucleus. Scale bar, 25 µm. Data were obtained from two independent cohorts. **d**, PC immunofluorescent staining (green, stained by ACIII) and PC length measurement in the CA3 region of mouse after stereotactic injections with either eSYN-shRNA-*Ift20-mir* or shRNA-scramble-mir. The length of the PC was measured and quantified in 3D using Imaris software. The length is expressed in µm, and 2–4 brain sections (*n* = 50–150 neurons per section) were analyzed per mouse. Scale bar, 10 µm. All data are expressed as mean ± s.e.m., and *P* values were determined by one-tailed (**a**) or two-tailed (**b**–**d**) Student’s *t*-tests compared to Scrb. Source data

We further investigated the link between core PC proteins and autophagy in hippocampal neurons. Downregulation of either *Ift20* or *Kif3a* decreases autophagy in the CA3 neurons, as shown by the reduction of LC3B puncta, together with the increase of SQSTM1/p62-positive puncta in comparison to controls (Fig. 3a). Of note, this decrease was not observed in dentate gyrus (DG) (Extended Data Fig. 3f). Similar results were obtained after *Ift20* downregulation in mature hippocampal neurons (AAV9-eSYN-shRNA-*Ift20*-mir) (Fig. 3b,c). This reduction in autophagy observed after neuronal *Ift20* postnatal downregulation is associated with longer PC axonemes (Fig. 3d).

### Core PC proteins influence learning and memory

**Fig. 4:**
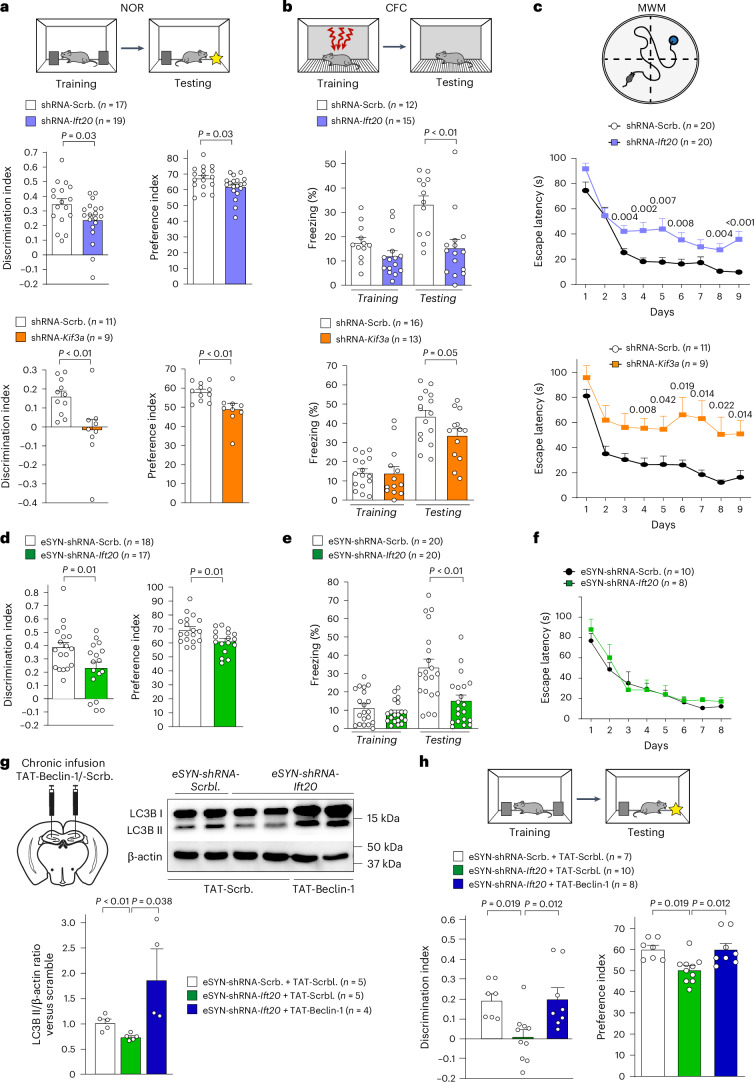
Selective downregulation of PC core proteins in hippocampal neurons leads to severe learning and memory impairments. **a**–**c**, Behavioral analyses performed in 3-month-old mice after hippocampal stereotactic injections with AAV-U6-shRNA-*Ift20*, AAV-U6-shRNA-*Kif3a* or their respective AAV-U6-shRNA-scramble. NOR test (**a**), CFC (**b**) and MWM test (**c**) were assessed 3 weeks after local AAV injections. For the NOR, discrimination and preference indexes were measured 24 h after the training phase to assess memory performances. For the CFC, the percentage of freezing was measured for the training and testing phases. For the MWM, the graph shows the time to localize a submerged platform in the swimming area. All behavioral tests were performed in two independent cohorts of animals for each group and their respective control. **d**–**f**, The same behavioral analyses (NOR (**d**); CFC (**e**); MWM (**f**)) were performed in 3-month-old mice, 3 weeks after hippocampal stereotactic injections with either eSYN-shRNA-*Ift20mir* (eSYN-shRNA-*Ift20*) or eSYN-shRNA-scramble-mir (eSYN-shRNA-Scrb.). This analysis was performed in two independent cohorts of animals for each group, for each behavioral test. **g**, Representative western blot and quantification of LC3B-II accumulation (LC3B-II/β-actin ratio) in mouse hippocampi stereotactically injected with either eSYN-shRNA-*Ift20-mir* or eSYN-shRNA-scramble-mir and after five consecutive days of daily infusion with either TAT-scramble or TAT-Beclin-1. β-actin was used as a loading control for each sample. The quantification is relative to eSYN-shRNA-scramble-mir mice injected with TAT-scramble. **h**, NOR performed in the same experimental conditions as Fig. 4a, in 3-month-old mice after hippocampal stereotactic injections with eSYN-shRNA-*scramble-mir* + TAT-scramble, eSYN-shRNA-*Ift20-mir* + TAT-scramble or eSYN-shRNA-*Ift20mir* + TAT-Beclin-1. This analysis was performed in one cohort of animals for each group. Data are expressed as mean ± s.e.m. *P* values were determined by one-tailed (**g**) or two-tailed (**a**, **b**, **d**, **e**) Student’s *t*-tests compared to shRNA-Scrb. The *P* values were determined by one-way (**h**) or two-way (**c**, **f**) ANOVA. Source data

**Extended Data Fig. 4:**
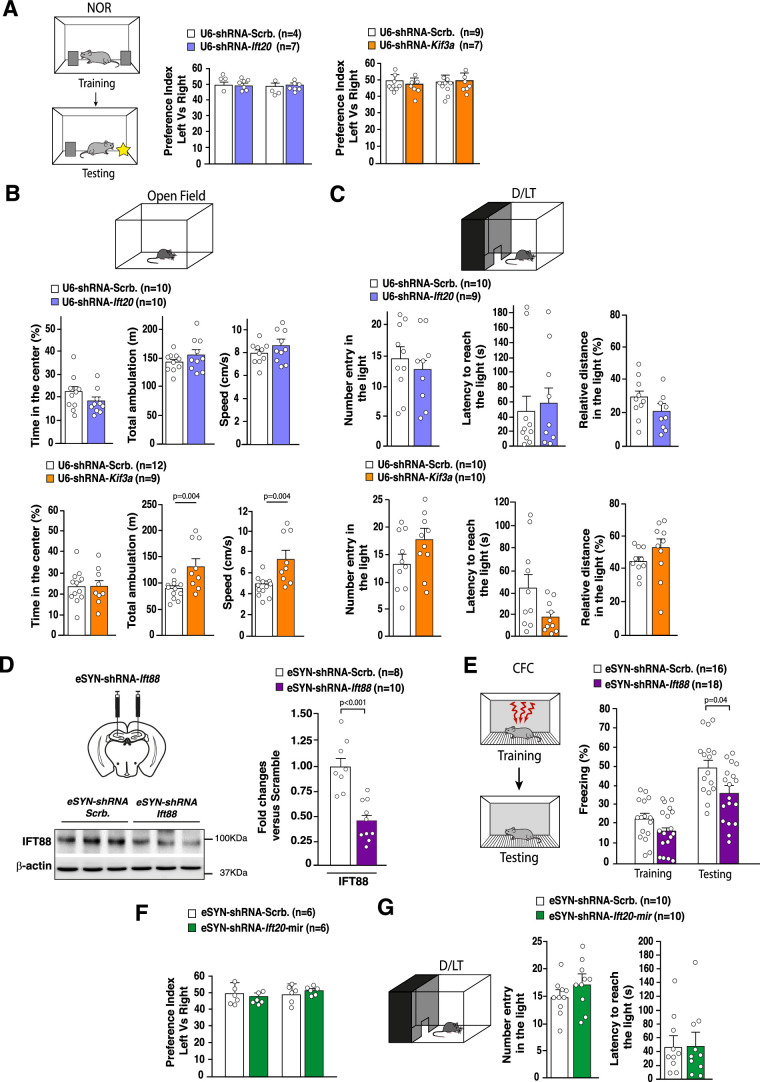
Hippocampal downregulation of core-PC proteins does not affect exploratory- and anxiety-like behaviors. **A**) As control, preference index for the (left versus right) object location during the training phase of the NOR was measured in 3-month-old mice, 3 weeks after hippocampal stereotactic injections with either AAV-U6-shRNA-Ift20, AAV-U6-shRNA-Kif3a, or their respective AAV-U6-shRNA-Scramble. The NOR was performed in two independent experiments for each group and its respective control. **B**-**C**) Open field test (OFT) (**B**) and Light-dark test (LDT) (**C**) performed in 3-month-old mice, 3 weeks after hippocampal stereotactic injections with either AAV-U6-shRNA-Ift20, AAV-U6-shRNA-Kif3a, or their respective AAV-U6-shRNA-Scramble. For the OFT, time spent in the center of the arena (%), total ambulation (meter), and speed (cm/s) were measured. For the LDT, number of entries in the light compartment, latency to reach the light box (seconds) and distance spent in the light box (%) were measured. These analyses were performed on 1 cohort of mice for each group. **D**) Representative western blot and quantification of IFT88 in mouse hippocampi, 3 weeks after stereotactic injections with either eSYN-shRNA-Ift88-mir or eSYN-shRNA-Scramble-mir. β-actin was used as a loading control for each sample. This analysis was performed on 2 independent cohorts of mice for each group. **E**) Contextual Fear Conditioning test (CFC) performed in 3-month-old mice, 3 weeks after stereotactic injections with either eSYN-shRNA-Ift88-mir or eSYN-shRNA-Scramble-mir. Percent freezing was measured for the training and testing phases. The CFC was performed in 2 independent cohorts of mice. **F**-**G**) Preference index for the (left versus right) object location during the training phase of the NOR (**F**), and number of entries in the light compartment and latency to reach the light box (seconds) during the Light-dark test (LDT) (**G**) performed in 3 month-old mice, 3 weeks after hippocampal stereotactic injections with either eSYN-shRNA-Ift20-mir or eSYN-shRNA-Scramble-mir. Data are expressed as mean ± SEM.The p values were determined by a two-tailed Student’s t-test compared to U6-shRNA-Scrb in B and C or to eSYN-shRNA-Scrb in D, E and G. Source data

We previously found that neuronal autophagy in the hippocampus is essential to foster neuronal homeostasis and, thereby, memory^20,35^. Therefore, we investigated the functional impact of decreasing the levels of hippocampal core PC proteins for the regulation of cognitive functions. To that end, we subjected our mouse models of hippocampal *Ift20 and Kif3a* downregulation to three independent behavioral tests assessing hippocampal-dependent learning and memory^53,54^: the novel object recognition (NOR) test, the three foot shocks contextual fear conditioning (CFC) paradigm and the Morris water maze (MWM) test (Fig. 4a–c). NOR and CFC tests assess associative memory and rely on two phases: a training phase, in which mice are exposed to novel memory stimulations (NOR: exposure to two similar objects; CFC: three foot shocks associated with a neutral environment) and a testing phase performed 24 h after the training phase, in which memory performances are evaluated (NOR: recognition of a novel exposed object; CFC: measurement of context-elicited freezing). In NOR, we found that *Ift20* or *Kif3a* downregulation impaired memory capacities, as the time spent to explore a novel object decreases compared to controls (Fig. 4a and Extended Data Fig. 4a). We confirmed these results in CFC, because mice downregulated for core PC proteins exhibited a decrease in freezing during contextual memory testing in comparison to controls (Fig. 4b). Spatial learning and memory were assessed using MWM, in which latency to locate the platform was recorded for each trial. Hippocampal downregulation of *Ift20* or *Kif3a* led to a decrease in memory performances, as shown by the increase in the latency to locate the escape platform in comparison to controls (Fig. 4c). By contrast, downregulation of *Ift20* or *Kif3a* did not affect performance in the open-field test (OFT) and the light/dark test (LDT), indicating that exploratory-like and anxiety-like behaviors are unaffected (Extended Data Fig. 4b,c). Notably, the downregulation of key core PC proteins in mature hippocampal neurons after stereotactic injections of AAV9-eSYN-*Ift20*-shRNAmir led to similar memory deficits, without affecting exploratory-like and anxiety-like behaviors (Fig. 4d,e and Extended Data Fig. 4f,g). Similar results were obtained with AAV9-eSYN-*Ift88*-shRNAmir (Extended Data Fig. 4d,e). By contrast, downregulation of *Ift20* in mature neurons did not induce any change in MWM (Fig. 4f), suggesting a potential implication of core PC proteins in neuronal progenitors, maturing neurons and/or other hippocampal resident cells (such as astrocytes and microglial cells). Taken together, these results demonstrate that reducing the levels of key core PC proteins in hippocampal neurons leads to memory impairments without affecting exploratory-like and anxiety-like behaviors.

### Autophagy induction counteracts cognitive deficits

To examine the functional role of the core PC-protein-dependent autophagy in the regulation of cognition, we tested whether inducing hippocampal autophagy could counteract the memory deficits induced upon neuronal downregulation of *Itf20*. To that end, we performed hippocampal daily infusion of either TAT-Beclin-1 peptide (pharmacological inducer of AP formation^55^) or TAT-scramble (control), after implantation of bilateral cannulas in *Ift20-*downregulated animals. We validated that TAT-Beclin-1 infusions improve the defects in autophagy observed after neuronal *Ift20* downregulation (Fig. 4g), as LC3B-II significantly accumulates upon TAT-Beclin-1 treatment. Then, we found that the improved level of hippocampal autophagy is sufficient to restore the recognition and contextual memory deficits induced by neuronal *Ift20* downregulation (Fig. 4h).

### Restoring core PC protein levels improves memory deficits

**Fig. 5:**
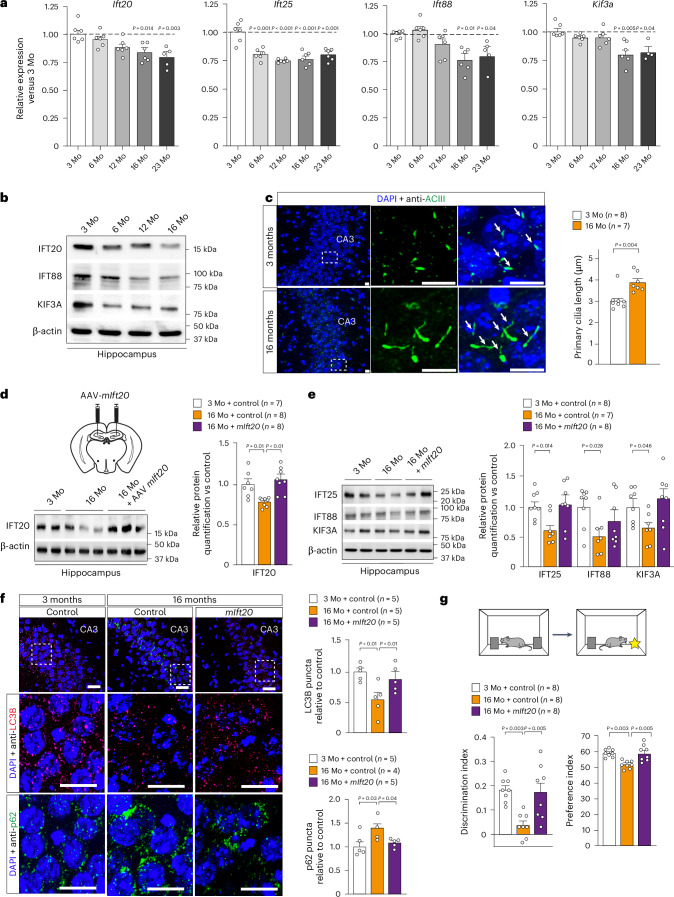
PC core protein levels are reduced during brain aging, and their restoration is sufficient to improve age-related memory decline. **a**, Relative expression of *Ift20*, *Ift25, Ift88* and *Kif3a* in WT dorsal mouse hippocampi at 3, 6, 12, 16 and 23 months of age. Quantification of mRNA expression is relative to the 3-month-old WT mouse group (*n* = 6 per group except for 23-month-old where *n* = 5 for *ift20*, *n* = 5 for *Ift88* and *n* = 4 for *Kif3A*). **b**, Representative western blot of IFT20, IFT88 and KIF3A protein levels in WT dorsal mouse hippocampi at 3, 6, 12 and 16 months of age. **c**, PC immunofluorescent staining (green, stained by ACIII) in the CA3 region of the hippocampus of young (3-month-old) and aged (16-month-old) mice. The length of the PC was measured and quantified in 3D using Imaris software. Scale bar, 5 µm. **d**, Western blot image and quantification of IFT20 protein in 3-month-old or 16-month-old mice, 3 weeks after stereotactic injections with either AAV-GFP (control) or AAV-m*Ift20* (expressing mouse *Ift20* cDNA). **e**, Western blot images and quantification of IFT25, IFT88 and KIF3A levels in the same experimental groups as in **d**. These measurements were performed in two independent experiments. **f**, LC3B and SQSTM1/p62 immunofluorescence (LC3B in red and p62 in green) and puncta quantification performed on brain cross-sections, at the level of the hippocampal CA3 region of 3-month-old or 16-month-old mice, 3 weeks after stereotactic injections with AAV-GFP (control) or AAV-m*Ift20* cDNA. Scale bars, 20 µm. **g**, NOR performed in 3-month-old or 16-month-old mice, after hippocampal stereotactic injections with AAV-GFP (control) or AAV-m*Ift20* cDNA. Discrimination and preference indexes were measured 24 h after the training phase to assess memory performances. The NOR was performed in two independent experiments for each group of mice. β-actin was used as a loading control for each sample in all western blot analyses. Data are expressed as mean ± s.e.m., and *P* values were determined by one-tailed (**f**) or two-tailed (**c**–**e**) Student’s *t*-tests or by one-way ANOVA (**a**, **g**). Mo, month-old. Source data

**Extended Data Fig. 5:**
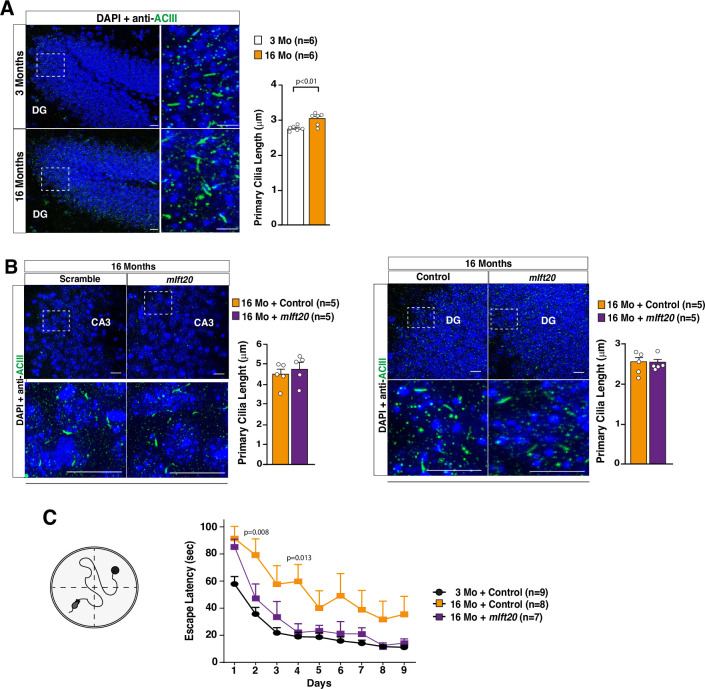
Restoration of neuronal IFT20 levels in old hippocampi reverses age-related cognitive deficits, without changing PC size. **A**) Primary cilia immunofluorescent staining (ACIII antibody) in the DG region of the hippocampus of from young (3Mo) and aged (16Mo) mice. The length of the primary cilia was measured and quantified in 3D using the Imaris software. Scale bar: 5 μm. **B**) Primary cilia length quantification obtained after immunofluorescent staining (ACIII) in the CA3 (left panel) and DG (right panel) regions of the hippocampus of 16 month-old mice, 3 weeks after local stereotactic injections with either AAV-Ift20 cDNA (mIft20) or AAV-GFP (Control). The length of the primary cilia was measured and quantified in 3D using the Imaris software. Scale bar: 10 μm. **C**) Morris water maze test (MWM) performed in 3- and 16-month-old mice, 3 weeks after local stereotactic injections with either AAV-Ift20 cDNA or AAV-GFP (Control). The MWM was performed in 1 cohort of mice. Data are expressed as mean ± SEM. The p values were determined by a two-tailed Student’s t-test compared to 3 Mo in A and by a Two-way ANOVA with multiple comparison in C. Source data

Normal brain aging is characterized by a decline in autophagy machinery in hippocampal neurons, leading to memory deficits^3,20^. Notably, we observed that this alteration is correlated to a marked decrease of core PC constituents (IFT20, IFT88 and KIF3A), at both the mRNA and protein levels (Fig. 5a,b) in aged (16-month-old) mouse hippocampi. This decrease is associated with PC morphological abnormalities, characterized by a longer PC axoneme in hippocampal neurons of both CA3 (Fig. 5c) and DG regions (Extended Data Fig. 5a). These morphological abnormalities are similar to those observed in PC after *Ift20* downregulation in hippocampal neurons of 3-month-old mice (Fig. 3d). Then, we tested whether restoring core PC protein levels in old hippocampi could improve age-related neuronal autophagy decrease and memory deficits. To do so, we performed stereotactic injections of an AAV9 expressing *Ift20* cDNA in aged hippocampi (Fig. 5d). Three weeks after the injections, we confirmed that the IFT20 level was restored in old hippocampi (Fig. 5d). This restoration was accompanied by an improvement of the levels of other core PC proteins, such as IFT25, IFT88 and KIF3A (Fig. 5e). Notably, restoring *Ift20* levels was sufficient to increase the LC3B levels and reduce SQSTM1/p62 accumulation in old hippocampi, suggesting an improvement of autophagy activity in the CA3 of aged mice (Fig. 5f). Exposing these mice to behavioral tests, we observed that the restoration of neuronal IFT levels in old hippocampi, despite no change in PC size, is sufficient to reverse age-related cognitive deficits (Fig. 5g and Extended Data Fig. 5b,c). Together, these results indicate that the reduction of core PC protein levels in the hippocampus during aging is associated with cognitive decline. Their restoration is sufficient to improve memory as well as neuronal autophagy decline in old hippocampi.

### PC-dependent autophagy mediates the OCN effects on cognition

**Fig. 6:**
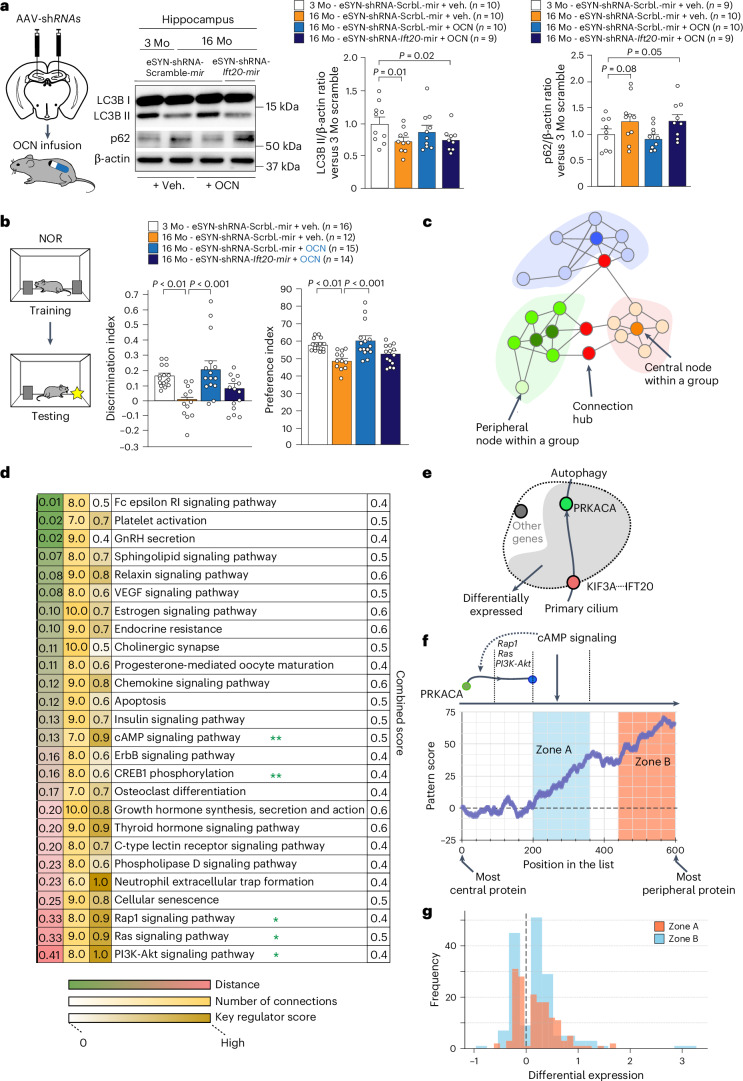
The PC core protein IFT20 is required to mediate the rejuvenating effects of OCN on age-related memory deficits. **a**,**b**, Stereotactic injections with AAV-*eSYN*-shRNA-scramble-mir or AAV-*eSYN*-shRNA-*Ift20-mir* were performed on 3-month-old or 16-month-old mice before 28-d peripheral infusion with vehicle or OCN. In **a**, western blot analysis and quantification of LC3B-I/II and SQSTM1/p62 accumulation from hippocampi. In **b**, discrimination and preference indexes of the NOR test. The NOR test was performed in two independent experiments for each group of mice. Data are expressed as mean ± s.e.m., and *P* values were determined by one-tailed Student’s *t*-test compared to control groups (**a**) and by one-way ANOVA with Tukey’s multiple comparison test (**b**). **c**, Graph theory and network analysis to determine elements within the network. Nodes represent pathways or protein expression. By analyzing connections between nodes, we can identify central and peripheral nodes within a cluster (or the entire network). Central nodes are associated with the core functionality of the cluster, whereas nodes positioned at the periphery of the cluster are associated with the nodes contributing to communication coordination. **d**, Pathway interaction graph based on the analysis of differentially expressed proteins from our proteomic data with KEGG databases, using a graph theory approach. Pathways were ordered based on their importance within the overall network using three parameters as detailed in the main text. Pathways with a combined coefficient greater than 0.35 are listed. *: pathways closely associated with those of zone A; **: pathways belonging to zone A. **e**, Graph illustrating the link between PC and autophagy-related pathways. A direct link could come through a KIF3A–PRKACA interaction. **f**, Identification of coordinated expression patterns within pathways in **d**. Only proteins with a significant impact within the network connectivity were taken into account. Rap1, Ras and PI3K/Akt pathways were the closest ones to localize near the identified zone A. Within the zone A, the most representative expressed proteins were involved in the cAMP signaling pathway. **g**, Histogram showing the strength of coordinated expression pattern in differentially expressed proteins from the ranked signaling pathway list in **d**. Mo, month-old. Source data

**Extended Data Fig. 6:**
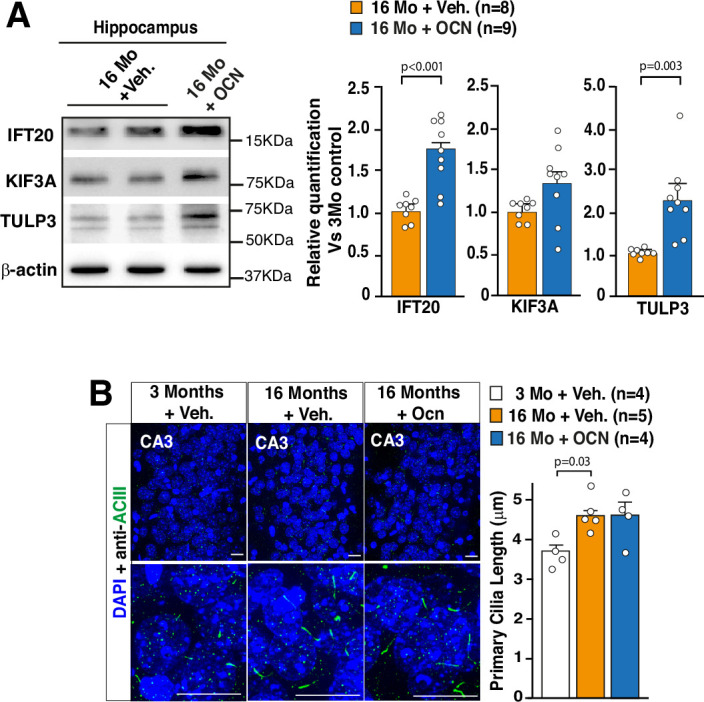
OCN treatment in old mice improves core PC-protein levels in hippocampus, without reversing PC length. **A**) Representative western blot image and quantification of IFT20, KIF3A, and TULP3 in 16- month-old mice 4 h after hippocampal stereotactic injection of either vehicle or OCN. β-actin was used as a loading control for each sample. The quantification is relative to Veh. injected group. **B**) Primary cilia length quantification obtained after immunofluorescent staining (ACIII antibody) in the CA3 (left panel) region of the hippocampus of 3 and 16- month-old mice after peripheral chronic infusion (peripheral osmotic minipumps) with either vehicle or OCN. The length of the primary cilia was measured and quantified in 3D using the Imaris software. Scale bar: 10 μm. Data are expressed as mean ± SEM. The p values were determined by a two-tailed Student’s t-test compared to veh in A and by a one-way ANOVA with multiple comparison followed by a Tukey test in B. Source data

These findings prompted us to test whether core PC proteins are required to transduce the OCN effects on age-related cognitive decline. We showed that a local stereotactic injection of recombinant OCN in 16-month-old mice is sufficient to improve the reduction of core PC proteins, namely IFT20 and TULP3, in the hippocampus (Extended Data Fig. 6a). Conversely, the improvement of autophagy enfeeblement and memory alterations in aged mice after systemic OCN administration is abolished after *Ift20* downregulation in hippocampal neurons (Fig. 6a,b). However, although OCN treatment in 16-month-old mice is able to improve core PC protein levels (Extended Data Fig. 6a), it is not sufficient to reverse the morphological abnormality in PC length (Extended Data Fig. 6b). Altogether, these data suggest a necessary contribution of core PC-protein-dependent autophagy to mediate the OCN’s effects in fostering cognitive fitness.

### cAMP signaling pathway modulates OCN-induced autophagy

We further explored the signaling pathway linking PC and induced autophagy upon OCN treatment. To do so, we used our proteomic data (Fig. 1a) that we further analyzed with public differential gene expression datasets coupled with Kyoto Encyclopedia of Genes and Genomes (KEGG) databases, using a graph theory approach (Fig. 6c–g). We elaborated three complementary parameters that we combined together to prioritize signaling pathways: (1) a distance-like measure that quantifies the pathway’s absolute divergence from autophagy by measuring the difference in how gene expression within the pathway contributes to the network’s overall structure; (2) strength of the connection between the pathway and autophagy; and (3) a key regulator score that describes the potential that the certain pathway could have the role of a regulator within the network. The pathways with combined coefficient greater than 0.35 were identified and ranked from the closest to the furthest pathway when compared to autophagy (Fig. 6d).

Among the signaling pathways selected, we revealed an interaction chain between core PC proteins (KIF3A linked to IFT20) - cAMP signaling (protein kinase cAMP-activated catalytic subunit alpha (Prkaca)) - autophagy (Fig. 6d,e). We further analyzed the patterns of differentially expressed proteins within the above-ranked pathways and revealed two distinct zones of interest (zones A and B). Zone A exhibited a more pronounced and stronger coordinated expression profile compared to zone B, as illustrated in the histogram profile of differentially expressed proteins (Fig. 6f). The enriched pathways the most closely associated with autophagy are the ones lying in front of those listed in zone A: Rap1, Ras and PI3K/Akt signaling pathways (Fig. 6d,f). Within the zone A, the most representative expressed proteins were those involved in the cAMP signaling pathway (Fig. 6d–g).

### OCN regulates autophagy via CREB signaling in hippocampus

**Fig. 7:**
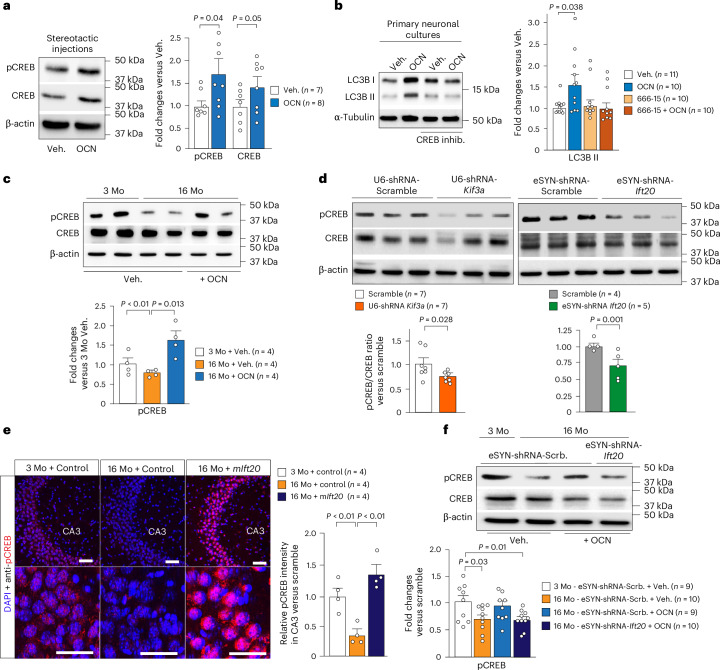
CREB signaling pathway is required to mediate the autophagy machinery regulation by OCN. **a**, Representative western blot and quantification of phospho-CREB and CREB protein levels in the hippocampus of 3-month-old mice, after stereotactic injections of vehicle or OCN. **b**, Representative western blot and quantification of LC3B-II accumulation (LC3B-II/β-actin ratio) in mature primary hippocampal neurons treated for 4 h with vehicle, OCN, CREB inhibitor (666-15) or OCN + CREB inhibitor. These measurements were obtained from two independent experiments. **c**, Western blot analysis and quantification of phospho-CREB and CREB in the hippocampus of 3-month-old and 16-month-old mice after stereotactic injections of vehicle or OCN. **d**, Western blot and quantification of phospho-CREB and CREB in 3-month-old mice previously injected in the hippocampus with AAV-*eSYN1-*shRNA-*Ift20*, AAV-*U6-*shRNA-*Kif3a* or AAV-U6/eSYN1-shRNA-scramble. **e**, Representative images of phospho-CREB immunofluorescence staining (red) performed at the level of the hippocampal CA3 region. Scale bar, 20 µm. Brain cross-sections of 3-month-old and 16-month-old mice collected 3 weeks after hippocampal stereotactic injections with either AAV-GFP (control) or AAV-m*Ift20* cDNA were used. Relative intensity quantification was measured relative to the 3-month-old mice injected with AAV-CMV-GFP. **f**, Western blot analysis and quantification of phospho-CREB and CREB in the hippocampus of 3-month-old and 16-month-old mice, after hippocampal stereotactic injections of either AAV-*eSYN*-shRNA-scramble-mir or AAV-*eSYN*-shRNA-*Ift20-mir* and peripheral chronic infusion with vehicle or OCN. The quantification is relative to 3-month-old mice injected with AAV-*eSYN*-shRNA-scramble-mir and treated with vehicle. These measurements were obtained from two independent cohorts of mice for each group. β-actin was used as a loading control for each sample in all western blots. Data are expressed as mean ± s.e.m., and *P* values were determined by two-tailed Student’s *t*-tests (**b**–**d**, **f**) or by one-way ANOVA followed by Tukey’s multiple comparison test (**e**). Mo, month-old. Source data

**Extended Data Fig. 7:**
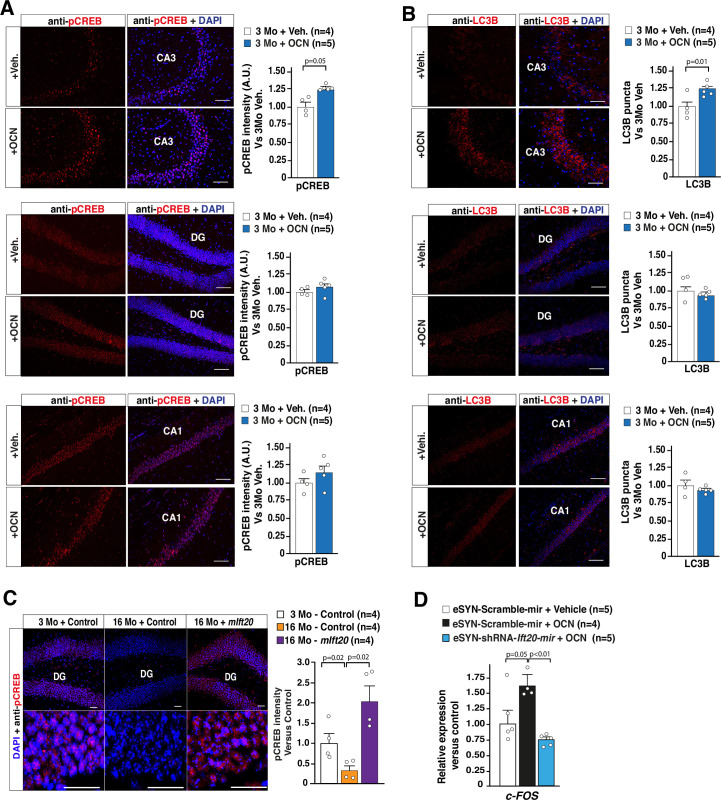
OCN enhances CREB phosphorylation and LC3B levels in the CA3 region of hippocampus. **A**-**B**) Phospho-CREB (**A**) and LC3B (**B**) immunofluorescence and quantification (relative intensity) performed on brain cross-sections (hippocampal CA3, Dentate Gyrus (DG) and CA1 regions) of 3-month-old mice 4 h after hippocampal stereotactic injection with either vehicle or OCN. Puncta analysis was performed using Icy Software. Scale bar: 50 μm. **C**) Phospho-CREB immunofluorescence and quantification (relative intensity) performed on brain cross-sections (hippocampal DG region) of 3- and 16-month-old mice, 3 weeks after local stereotactic injections with either AAV-GFP (Control) or AAV-mIft20 cDNA. The analysis was performed in 1 cohort of mice. Scale bar: 20 μm. **D**) Relative gene expression of c-Fos in the hippocampi of 3-month-old mice previously injected in the hippocampus with either AAV-eSYN-shRNA-Ift20mir or eSYN-shRNA-Scramble-mir. The mice were subjected to stereotactic injection of either vehicle or OCN. Data are expressed as mean ± SEM. The p values were determined by a one-tailed (**A**, **B**, **D**) or a two-tailed (**C**) Student’s t-test. Source data

It is well described that activation of the transcription factor cAMP response element-binding protein (CREB) is decreased in aged mouse hippocampi^54–56^. These alterations in CREB signaling contribute to cognitive deficits in normal aging and various neuro-degenerative diseases^53–55^. Multiple reports showed that the rejuvenating effects of the young plasma administration on cognition are mediated, in part^57,58^, through the activation of CREB phosphorylation^9,13^. Moreover, we found that the cAMP signaling pathway is among the closest autophagy-related pathways regulated upon OCN treatment (Fig. 6d). Western blot and immunofluorescence analyses showed that OCN treatment is sufficient to enhance CREB phosphorylation and LC3B levels in the CA3, when no significant differences were observed in the CA1 and DG regions (Fig. 7a and Extended Data Fig. 7a,b).

Accordingly, we showed that the increase in LC3B-II accumulation in primary hippocampal neurons upon OCN is abolished after CREB inhibitor (666-15) treatment (Fig. 7b), suggesting that the CREB signaling pathway is required for the regulation of OCN-induced autophagy in hippocampal neurons. We next confirmed that CREB phosphorylation is lower in 16-month-old hippocampi and that this decrease could be reversed after OCN stereotactic injections (Fig. 7c). Hippocampal downregulation of either *Ift20* or *Kif3a* decreased neuronal pCREB/CREB ratio (Fig. 7d). Conversely, the restoration of IFT20 levels (after local AAV9-*Ift20* cDNA injections) is sufficient to improve CREB phosphorylation deficits in the CA3 and DG of 16-month-old mice (Fig. 7e and Extended Data Fig. 7c). Moreover, we found that neuronal *Ift20* downregulation in the hippocampus is sufficient to counteract the induction of *c-FOS* expression, a major transcriptional target of pCREB, by OCN (Extended Data Fig. 7d). Downregulation of neuronal *Ift20* blocked the improvement of CREB phosphorylation by OCN in old mice (Fig. 7f). Altogether, these data support the necessary contribution of PC-dependent CREB signaling pathway to mediate the induction of autophagy machinery by OCN and, thereby, to the rejuvenating effects of OCN on age-related cognitive decline.

## Discussion

In our study, we identified that a core PC protein–autophagy machinery axis could act as a gateway in hippocampal neurons to transmit the effects of blood factors (OCN) on neuronal homeostasis and, thereby, allow for the maintenance of cognitive fitness. We found that GPR158 is present at the PC and that OCN can enhance the core PC protein levels in hippocampal neurons. By contrast, downregulation of core PC proteins is sufficient to block the induction of hippocampal autophagy machinery by OCN. Altogether, these data led us to test the functional relevance of the link between core PC proteins and autophagy in hippocampal neurons and as mediators of the effects of systemic factors (OCN) in cognitive fitness. Accordingly, we demonstrated that downregulation of core PC proteins in hippocampal neurons leads to reduced autophagy and memory performance. Restoring autophagy is sufficient to counteract memory deficits of these mice. During aging, core PC protein levels are reduced, which might be correlated to a PC dysfunction in this region of the brain. In fact, we showed that restoring their levels improves age-impaired autophagy and memory in old mice. By contrast, core PC protein downregulation abolished the pro-cognitive effects of OCN in aged mice. Last, we found that the core PC proteins transduce the activation of autophagy by OCN via CREB phosphorylation in hippocampal neurons, a key transcriptional factor for the control of neuronal plasticity and memory. These results indicate that PC-dependent neuronal sensing is impaired during aging, resulting in the inability to sense and transduce signals from systemic factors, such as OCN, to maintain neuronal homeostasis, autophagy and, therefore, learning and memory.

As previously demonstrated, mice exhibited a significant impairment of memory and autophagy machinery from 16 months of age, compared to 3-month-old mice, indicating the progressive feature of aging^18,20^. Chronic peripheral treatment with OCN in aged mice rescued age-related memory impairments, from 16 months of age^18,20^. However, the molecular mechanisms of this OCN-dependent rescue were not well understood, despite its therapeutic potential. We previously reported that OCN controlled age-induced memory impairment through autophagy^20^. Additionally, recent works have shown that autophagy regulation, among different factors, is downstream of the PC^36,37,59^. Although it is important to note that all our in vivo experiments were performed using male mice, our results suggest that the pro-cognitive effects of OCN are reduced when core PC proteins are downregulated in neuronal PC.

Exploring the signaling pathway linking PC and induced autophagy upon OCN, we used a bioinformatic approach, based on a graph theory analysis and differential gene expression data coupled with the KEGG database. We revealed an interaction chain between core PC proteins (KIF3A and IFT20) - cAMP signaling - autophagy. These data reinforce the link between PC and autophagy and solidify further the importance of the cAMP signaling pathway in the OCN-dependent induction of autophagy in hippocampal neurons.

A correlation was shown between autophagy induction and activation of CREB phosphorylation to mediate neuronal survival and protection^60,61^. Here, we showed that the effects of OCN on autophagic pathway are dependent on phospho-CREB, whose activation is reduced after core PC protein silencing. Notably, we demonstrated that restoring core PC proteins in aged mice is necessary and sufficient to correct the age-related phospho-CREB decline. Consistently, OCN treatment in aged mice requires core PC proteins to restore CREB activity. These data indicate that PC-dependent autophagic response is part of an integrated survival signaling network that activates the CREB pathway.

PC function as cellular antenna, enriched in specific GPCRs sensing and responding to neuromodulators in the extracellular milieu, being key in the transduction of the extracellular signals to regulate memory maintenance. Here, we showed that GPR158 localizes at the PC, where it interacts with PC proteins and transduces the OCN-mediated signal to regulate autophagy. Although the detailed molecular mechanism by which OCN boosts autophagy has not been detailed yet, we demonstrated the role of the core PC proteins in maintaining cognitive resilience.

In this study, we focused on the role of a PC–autophagy axis in neurons, but we cannot exclude that this axis could also play a role in other resident cell types in the brain. Indeed, OCN could also play a critical role for the myelination of oligodendrocytes through its binding to GPR37 (ref. ^62^). Therefore, one can ask whether this axis could also be activated and mediate the OCN effects in this specific cell type. Lastly, beyond OCN, it is known that numerous receptors for systemic factors are present in the PC^63^ and could modulate the autophagy process. Our findings may suggest that this PC–autophagy axis could constitute a key mechanism on how neurons communicate with the systemic milieu and enhance their adaptive response to environmental changes.

During aging, we found a reduction of key core PC protein levels in mouse hippocampi. This reduction is associated with a marked alteration of the transcriptional levels of genes encoding these core PC proteins. However, at the present time, it is difficult to determine whether the decline in core PC proteins is only due to transcriptional reduction or could be also associated with an age-related increase in core PC protein degradation. Further analyses are now needed to address this question and assess the degradative dynamics of these proteins in hippocampal neurons.

We showed that the structure of hippocampal neuronal PC is affected by aging, characterized by increased length. This result is consistent with the increased cilia length, but constant number of PC, observed in aged hippocampal neurons of rats^64^. The importance of an optimal cilia length is underscored by the finding that its perturbation, generating shorter or longer cilia, can be observed in cilia-related diseases. In such cases, the location or trafficking of PC-localized receptors or additional scaffold proteins can be impaired, resulting in disruption of PC-dependent signaling cascades, such as those induced by OCN. We demonstrated that the increase in PC length, together with a decrease in core PC proteins, is associated with poor memory performance. These data suggest the importance of PC turnover in the maintenance of cognitive functions. This result is also consistent with previous findings showing the involvement of PC in cognition, as the disruption of cortical and hippocampal PC promotes alterations in behavior, learning and memory^42,43^. Consistently, a recent study observed a decrease in ciliary genes in a mouse model of memory consolidation and demonstrated that failure to form lasting memories is accompanied by downregulation of PC-associated genes^65,66^. However, although we found that OCN injections or restoring IFT levels could correct age-related autophagy and memory decline, it is not sufficient to modify neuronal PC length in old mice. This observation may suggest that the age-related morphological PC alterations are irreversible and, consequently, that the PC morphology does not necessarily determine its functionality. Further experiments are needed to test whether prolonged OCN treatment could reverse this morphological defect or at least block the increase of PC length during the course of aging.

Neuronal sensing can be affected by age-related changes in systemic factor levels but also by dysfunctional detection of these hormones/signals, which finally impair signal transduction. Indeed, decrease in core PC proteins results in an incapacity to perceive systemic factor changes and induce the required signaling pathways, including autophagy, to modulate learning and memory behavior. Thus, a functional PC is required to integrate the pro-cognitive effects of OCN in aged mice. In young adult mice, OCN fails to promote neuronal hippocampal autophagy in a context of cilia impairment. Consistently, the OCN’s pro-cognitive effects, dependent on autophagy^20^, were deteriorated when core PC proteins were downregulated in neuronal PC. Despite that, we observed a partial amelioration of memory performances in OCN-treated mice with impaired IFT20, which can be attributed to the binding of OCN to non-ciliary-located GPR158 and IFT-independent functions.

Our study unravels a physiological role of the autophagy–PC axis in brain, where alteration of ciliary core PC proteins during aging leads to autophagy impairments and, thereby, memory deficits. This study identifies a paradigm in the mechanism controlling the communication between blood factors and neurons and its importance during brain aging for the maintenance of neuronal homeostasis in the hippocampus. PC could constitute a gateway in hippocampal neurons through which systemic factors modulate neuronal autophagy and, thereby, cognitive resilience. By shedding light on this regulatory mechanism responsible for the decline of autophagy activity during aging, our findings provide the foundation for the development of more potent therapeutic targets to treat or even prevent age-related cognitive disorders as well as neurological disorders in ciliopathies.

## Methods

### Animals

All experiments were performed on C57BL/6J WT male mice (Janvier Laboratory). All mice were 3 months or 16 months of age at the start of experiments, and littermates were used as controls. Upon arrival, mice were housed at least 2 weeks before any behavioral or molecular testing. Male mice were housed five animals per cage in polycarbonate cages (35.5 × 18 × 12.5 cm), under a 12-h light/dark cycle with ad libitum access to food and water before experimentation. All behavioral experiments were performed in accordance with the European Communities for Experimental Animal Use (2010/63/EU) and ethical committee review procedures and protocols (APAFIS- 25139).

### Proteomic analysis

Hippocampi lysates were prepared using BeatBox Tissue Kit 24x (PreOmics): samples were homogenized in 5% SDS 50 mM TEAB lysis buffer for 10 min, applying the high BeatBox setting. After 5-min heating at 95 °C and 10-min centrifugation at 14,000*g*, protein dosage was performed using a DC Protein Assay Kit (Bio-Rad). S-TrapTM micro spin column (ProtiFi) digestion was performed on 30 µg of cell lysates according to the manufacturer’s instructions. After elution, peptides were vacuum dried and resuspended in 75 µl of 2% acetonitrile (ACN) and 0.1% formic acid in high-performance liquid chromatography (HPLC)-grade water before mass spectrometry (MS) analysis. Then, 400 ng of each sample was injected on a nanoElute2 HPLC system coupled to a timsTOF Pro (Bruker Daltonics) mass spectrometer. HPLC separation (solvent A: 0.1% formic acid in water; solvent B: 0.1% formic acid in ACN) was carried out at 250 nl min^−1^ using a packed emitter column (C18, 25 cm× 75 μm, 1.6 μm) (Ion Optics) using a 70-min gradient elution (2% to 13% solvent B during 42 min; 13% to 20% during 23 min; 20% to 30% during 5 min; 30% to 85% for 5 min; and finally 85% for 5 min to wash the column).

Mass spectrometric data were acquired using the parallel accumulation serial fragmentation (PASEF) acquisition method in data-independent acquisition (DIA) mode. The measurements were carried out over the *m*/*z* range of 100 Th to 1,700 Th. The range of ion mobility values was from 0.6 Vs cm^−2^ to 1.6 Vs cm^−2^ (1/K_0_). diaPASEF settings were as follows: mass range from 400 *m*/*z* to 1,200 *m*/*z*, mobility range from 0.60 Vs cm^−2^ to 1.43 Vs cm^−2^ (1/K_0_), cycle time estimate of 1.79 s and 16 PASEF scans × two steps per PASEF scan (32 windows).

Data analysis was performed using DIA-NN software (version 1.8.1). A search against the UniProtKB/Swiss-Prot *Mus musculus* database (release July 2023, 17,167 entries) was performed using library-free workflow. For this purpose, ‘FASTA digest for library free search/library generation’ and ‘Deep learning spectra, RTs and IMs prediction’ options were checked for precursor ion generation. One trypsin missed cleavage was allowed, and the maximum variable modification was set to 2. Carbamidomethylation (Cys) was set as the fixed modification, and protein N-terminal methionine excision, methionine oxidation and N-terminal acetylation were set as variable modifications. The peptide length range was set to 7–30 amino acids, precursor charge range 2–4, precursor *m*/*z* range 300–1,300 and fragment ion *m*/*z* range 300–1,300. To search the parent mass and fragment ions, accuracy was set to 10 ppm manually. The false discovery rates (FDRs) at the protein and peptide level were set to 1%. Match between runs was allowed. For the quantification strategy, Robust LC (high precision) was used, whereas default settings were kept for the other algorithm parameters.

### Bioinformatic analyses

#### Identification of the most relevant autophagy-related enriched pathways associated with OCN treatment

Using custom-made scripts in Python and R languages, we constructed a gene co-expression network using a graph theory approach to analyze the network topology of genes in the hippocampus after OCN treatment. Leveraging the KEGG database, we built an initial network where nodes represented genes and/or pathways. We then enriched this network by retaining only statistically significant differentially expressed genes (adjusted *P* < 0.05). This resulted in a focused gene interaction map encompassing pathways relevant to our investigation.

We assessed how genes interact by looking at their connectivity within the network. We systematically removed the genes from the network components and measured how much the network’s connectedness dropped. Genes causing a larger decrease were considered more central and crucial. Then, we ordered the genes based on their importance within the overall network structure (starting with the most important ones). From this list, only central ones (genes with negative scores) were chosen and fed to g:Profiler where the ordered query was run. Genes with a positive score had an increase of centrality upon their removal, meaning that the graph becomes more connected, thus indicating that these genes were positioned at the periphery of the obtained network. Finally, we constructed a network that specifically highlights how these important genes interact within the identified pathways.

To identify pathways functionally linked to autophagy, we employed a combined approach. Pathway impact on network structure and distance from autophagy (based on connectivity change) were assessed. We then evaluated pathway centrality (considering both connections and connected neighbors) to create a ‘key regulator indexʼ. Finally, a coefficient integrating connection strength with autophagy, network impact similarity and the key regulator index was used to prioritize the most relevant autophagy-connected pathways.

#### Investigating the interactions of PC and autophagy

To investigate the interaction between PC and autophagy, the initial gene pathway map, enriched with data from biological experiments, was used. Next, the minimum node distance between the genes known to be part of the PC (obtained from the KEGG pathway database - PC and associated proteins) and autophagy signaling pathway was identified.

#### Integrating ordered gene list information with gene expression data

The patterns of activity were determined using the initial ordered list of genes. We introduced a score that integrated both the information about the differential expression and the topographical importance of the node within the network. We iterated the initial ordered list of genes and either incremented the position-specific score by +1 if the current gene of interest was upregulated or decremented it by −1 if downregulated. Values around a constant value indicated a random distribution of pattern expression data within a certain region of the list, whereas sustained positive/negative slope of the score highlighted the regions of topographically related genes with coordinated expression levels. These hubs offered a more comprehensive picture of a network’s functioning and likely represented a tightly controlled process, which was likely essential for the captured cellular response.

### Western blot analysis

Mouse dorsal hippocampi were dissected, snap frozen and lysed in RIPA lysis buffer with protease and phosphatase inhibitors. The lysates were sonicated, and the protein concentration was analyzed by BCA assay. The lysates were loaded on a 12.5% or 10% SDS polyacrylamide gradient gel and transferred onto a PVDF membrane. The blots were blocked in Tris-buffered saline with 0.1% Tween (TBST) and 5% BSA and incubated with the following: mouse anti-β-actin (1:5,000; Sigma-Aldrich, A-2228), rabbit anti-IFT20 (1:1,000; Proteintech, 13615), rabbit anti-IFT88 (1:1,000; Proteintech, 13967), rabbit anti-KIF3A (1:1,000; Proteintech, 13930), rabbit anti-IFT25 (1:1,000; Proteintech, 15732), rabbit anti-TULP3 (1:1,000; Proteintech, 13637), rabbit anti-GPR158 (1:1,000; Antibodies Online, ABIN6258978), mouse anti-Beclin-1 (1:1,000; BD Transduction Laboratories, 612113; only the top band corresponding to non-cleaved Beclin-1 was quantified in this study), rabbit anti-LC3B (1:10,000; Sigma-Aldrich, L7543), guinea pig anti-p62 (1:2,000; PROGEN, GP62-C), rabbit anti-phospho-CREB (1:1,000; Merck Millipore, 06-519) and rabbit anti-CREB (1:1,000; Cell Signaling Technology, 9197). Horseradish peroxidase (HRP)-conjugated secondary antibodies (anti-mouse IgG (Cell Signaling Technology, 7076), anti-rabbit IgG (Cell Signaling Technology, 7074) and anti-guinea pig IgG (Sigma-Aldrich, A5545)) were used for protein detection and revealed by Clarity Western ECL Substrate (Bio-Rad) or Immobilon Chemiluminescent HRP Substrate (Millipore). Selected films were scanned and quantified using Bio-Rad ChemiDoc Touch Imaging System version 1.0 and Image Lab software version 5.2, respectively. β-actin bands were used for normalization.

### Cell isolation and culture

Murine neuroblastoma N2a cells (American Type Culture Collection, Neuro-2a-CCL-131) were cultured in DMEM supplemented with 10% FBS and 100 U ml^−1^ penicillin–streptomycin. N2a cells were treated for 4 d with 10 µM retinoic acid to induce differentiation before treatment.

Hippocampal neurons were isolated from mouse embryos (embryonic day 16.5). After dissection, hippocampi were digested for 15 min at 37 °C into 0.05% trypsin and 0.02% EDTA. After three washes with DMEM (Thermo Fisher Scientific, 6196505) supplemented with 10% FBS, 100 U ml^−1^ penicillin–streptomycin and 1× GlutaMAX (Thermo Fisher Scientific), cells were dissociated by pipetting up and down and plated onto poly-l-lysine-coated plates or glass coverslips for microscopic examination. Twenty-four hours after plating, the media were replaced with Neurobasal medium (Thermo Fisher Scientific) containing B27 supplement (Thermo Fisher Scientific), GlutaMAX and MycoZap (Lonza) and refreshed twice a week. To decrease the proportion of glial cells, 1 µM Ara-C inhibitor (Sigma-Aldrich, C1768) was added in the pre-warmed complete Neurobasal medium, starting at day in vitro 5 (DIV5). Experiments were performed on cells after DIV15.

All cells were grown at 37 °C in a humidified incubator with 5% CO_2_.

### Neuronal stimulation treatment of primary hippocampal neurons

Before treatments, neurons were starved for 4 h (Neurobasal medium without B27). Neurons were treated for 5 min or 4 h with Neurobasal medium containing 10 ng ml^−1^ OCN. For the 4-h treatment, neurons were treated with Neurobasal medium containing 10 ng ml^−1^ OCN, 100 mM Baf or both. For experiments using CREB inhibitor, neurons were treated for 4 h with Neurobasal medium containing 10 ng ml^−1^ OCN, 73 nM CREB inhibitor 666-15 (Sigma-Aldrich, 5383410001) or both. After treatments, neurons were rinsed in PBS and proteins extracted in 1× Laemmli buffer containing phosphatase and protease inhibitors or fixed for 20 min in 4% paraformaldehyde (PFA)/4% glucose at room temperature.

### Recombinant OCN

Mouse uncarboxylated OCN was purified from BL21 bacteria transformed with pGEX2TK-mOCN as previously described^17,18^. In brief, GST–OCN fusion protein was bacterially produced in BL21 pLyS transformed with pGEX-2TK-mOCN after induction with IPTG. Bacteria were collected in lysis buffer (PBS, 10 mM Tris, pH 7.2, 2 mM EDTA, 1% Triton and 1× protease/phosphatase inhibitor cocktail; Thermo Fisher Scientific, 78443). After four freeze–thaw cycles and sonication, lysates were cleared by centrifugation. The supernatant was incubated for 4 h with Glutathione Sepharose 4B (GE Healthcare, 17075601) at 4 °C. After six washes with washing buffer (PBS 1× and 1% Triton) and PBS, OCN was cleaved out from the GST moiety by using thrombin (GE Healthcare, 27-0846-01). Four fractions were collected, and each of them was incubated for 30 min with Benzamidine Sepharose (GE Healthcare, 17-5123-10) at room temperature to remove thrombin. Then, 10 ng of OCN (diluted in PBS) was injected in mouse hippocampi. Primary hippocampal neurons were treated with OCN with 10 ng ml^−1^ in culture medium.

### Co-immunoprecipitation

We followed the manufacturer’s protocol (Life Technologies, Dynabeads Protein G-10007D) for TULP3 co-immunoprecipitation. N2a cell lysates were prepared with RIPA and protease/phosphatase inhibitors as described above and sonicated, centrifuged and analyzed for protein concentration by BCA assay. We linked the rabbit anti-TULP3 antibody (1 µg; Proteintech, 13637) or control rabbit IgG (1 µg; Proteintech, 30000-0A) to magnetic beads by incubation on a rotatory wheel for 30 min at room temperature, washed and incubated with 400 µg of protein extract overnight at 4 °C on a rotatory wheel. After three washes, we eluted the proteins, added loading buffer and DTT, denatured at 95 °C and resolved by western blot.

### Immunostaining on cross-sections and quantification

Mice were deeply anesthetized with a mixture of ketamine and xylazine and transcardially perfused with cold PBS, followed by cold 4% PFA. Brains were post-fixed overnight in 4% PFA at 4 °C. Then, 30-μm serial coronal floating sections were obtained using a vibratome. Immunofluorescence experiments were performed on sections blocked for 1 h in 0.3% Triton X-100 and 10% donkey serum in TBS at room temperature and then incubated overnight at 4 °C with guinea pig anti-p62 (1:200; PROGEN, GP62C), mouse anti-NeuN (1:400; Sigma-Aldrich, MAB377), rabbit anti-LC3B (1:200; Sigma-Aldrich, L7543), rabbit anti-pCREB (1:500; Merck Millipore, 06-519) and rabbit anti-ACIII (1:400; Thermo Fisher Scientific, PA5-35382) diluted in 5% normal donkey serum in TBS–0.1% Tween.

For in vitro immunostaining, cell coverslips were permeabilized for 15 min with 0.2% Triton X-100 and then blocked for 30 min with 3% BSA at room temperature before being incubated with rabbit anti-TULP3 (1:100; Proteintech, 13637-1-AP), rabbit anti-ACIII (1:400; Thermo Fisher Scientific, PA5-35382) and mouse anti-GPR158 (1:100; Bio-Techne, MAB10286) overnight at 4 °C.

Sections and coverslips were washed with TBS–0.1% Tween before and after being incubated for 2 h with secondary antibodies (donkey anti‐guinea pig IgG (1:200; Bioss, Alexa Fluor 555), donkey anti‐rabbit IgG (1:200; Bioss, Alexa Fluor 647), donkey anti‐rabbit IgG (1:200; Bioss, Alexa Fluor 546), donkey anti‐mouse IgG (1:200; Bioss, Alexa Fluor 488) or donkey anti‐rabbit IgG (1:200; Jackson ImmnuoResearch, Cy3)) at room temperature in TBS–0.15% Triton. After PBS washes, Hoechst 33342 was used to stain nuclei (1:5,000; Thermo Fisher Scientific, 62249) for 5 min at room temperature. All sections and coverslips were mounted onto gelatin-subbed slides using Fluoromount G (Sigma-Aldrich). Images were obtained using a Zeiss Apotome 2 fluorescence microscope and analyzed using Zeiss ZEN lite LSM software under the same setting. The number of cells with SQSTM1/p62 puncta was quantified on digital images with Icy software (http://icy.bioimageanalysis.org). For phospho-CREB intensity quantification, images were acquired from five hippocampal sections of the different groups and transformed to a grayscale, and the signal was analyzed by calculating the integrated density of a region of interest after defining a threshold with ImageJ software (National Institutes of Health; available at http://rsb.info.nih.gov/ij/).

### PC counting and analysis

Cilia length analysis was performed on the microscopy analysis Imaris software program version 9.2.0 (Bitplane). Apotome images of the CA3 and DG hippocampal regions were imported and first converted to the Bitplane format. PC were counted using Imaris FilamentTracer. Bitplane images were analyzed by manually tracing over PC using the Pencil tool in AutoPath mode. The start of the cilium was determined as the start of the red signal to the end of the same elongated signal. Cilia were traced only where Hoechst dye was apparent. Imaris functionality provided automatic centering of traced cilia once a cilium was delineated manually. Cilia were traced in 3D. Imaris statistics of cilia length, number of cilia per image and cilia straightness were then exported to Excel files for later data analysis. The number of cilia per image was normalized by dividing the number of cilia by the volume of the nuclei (μm^3^). The area was delimited by using the built-in Imaris surface tool. The detection of voxels threshold was manually adjusted to either increase or decrease coverage of nuclei that needed to be included in the volume of calculation. Between 49 and 116 cilia were examined for each image in the totality of the CA3 region, and between 85 and 368 cilia were examined for the upper layer of the DG. Three to four images were analyzed per mouse per region per age in the different experiments.

### GPR158 quantification and analysis

GPR158 analysis within the PC was performed on the microscopy analysis software program Imaris (version 9.2.0; Bitplane). Apotome images of the primary hippocampal neurons were converted to the Bitplane format. PC were first outlined with Imaris FilamentTracer in 3D, and Bitplane images were analyzed by manually tracing over PC using the Pencil tool in AutoPath mode following the same method as described above. The localization of GPR158 was then measured in 3D using the Spot Detection tool. We performed an automatic segmentation of objects, based on GPR158 signal intensity, with an estimated diameter of the spots of 0.280 µm. The Model PSF-elongation along the *z* axis and background subtraction options were selected. The Quality filter was manually adjusted to 57.6 on all images, so that the spots fit with GPR158 signal intensity. In the Spots menu, the filter ‘Shortest distance to Cilia #’ was added, and this process was repeated for all the cilia of the image. Then, the different distances to the cilia were set up (from 0 µm to 10 µm from the PC), and the number of GPR158 spots was read for all the distance groups.

### Semi-quantitative RT–PCR

Brain tissues were flash frozen after dissection, and total RNA was isolated with TRIzol reagent using a homogenizer. Single-stranded cDNA was synthesized from total RNA (2 μg) by using SuperScript II Reverse Transcriptase. qRT–PCR was performed using iTAQ SYBR Green (Bio-Rad). The following primers were used: *Ift20* primers (5′-AGGCAGGGCTGCATTTTGAT-3′ and 5′-CAAGCTCAATTAGACCACCAACA-3′), *Ift88* primers (5′-ACAGTGGCCAGAACAATAGTG-3′ and 5′-TCATCCTCGTCAATTCTTTTC-3′), *Kif3a* primers (5′-ATGCCGATCAATAAGTCGGAGA-3′ and 5′-GTTCCCCTCAT TTCATCCACG-3′), *Ift25* primers (5′-TGATTCTGGCCACATCGAGTGATGA-3′ and 5′-TCCTGGGGAAACATGCCTGTGG-3′), cFos primers (5′-CAGAGCGCAGAGCATCGCCA-3′ and 5′-CGATTCCGCCACTTGGCTGC-3′) and *Gapdh* primers (5′-AGGTCGGTGTG AACGGAT-3′ and 5′-GGGGTCGTTGATGGCAACA-3′). Data were analyzed with CFX Maestro software version 1.1.

### Stereotaxic injections and hippocampal cannula implantations

Mice were anesthetized by intraperitoneal injection of ketamine hydrochloride (100 mg kg^−1^) (1000 Virbac) and xylazine (10 mg kg^−1^) (Rompun 2%; Bayer) and placed in a stereotaxic frame (Kopf, 900SL). Ophthalmic eye ointment was applied to the cornea to prevent desiccation. The area around the incision was trimmed, and Vetoquinol was applied.

For the stereotactic injections, all drugs were administrated bilaterally into the dorsal hippocampi (coordinates from bregma; Paxinos and Franklin (2008): *x* = ±1.4 mm, *y* = 2.0 mm and *z* = −1.34 mm). Then, 1-μl volume of either AAV or drugs was injected over 4 min (injection rate, 0.25 μl min^−1^). To limit reflux along the injection track, the needle was maintained in situ for 4 min between each 1-μl injection. At the end of the surgical procedure, the skin was stitched over the brain, and an analgesic solution was administered subcutaneously (ketoprofen, 5 mg kg^−1^).

For the cannula implantation, a hole was made at the following coordinates: anterior-posterior (AP) −2.0, medial lateral (ML) −1.4 (in mm, from bregma). A guide cannula (Plastics One, C315GMN/1.5, 26 gauge) was secured to the skull with dental cement (Sun Medical, Super-Bond C&B). Lastly, the scalp incision was sutured around the cannula, and an analgesic solution was administered subcutaneously (ketoprofen, 5 mg kg^−1^).

### Drug preparation and hippocampal cannula infusion

TAT-Beclin-1 or TAT-scramble was dissolved to a concentration of 1 μg μl^−1^ in PBS. For 7 d, the experimenter gently restrained the mouse and placed an injector cannula (C315IMN/1.5/0.5; 33 gauge, extending 0.5 mm beyond the 1.5-mm guide cannula). The drug was infused (1.0 μl per side at a rate of 1 μl min^−1^) using a syringe pump (Harvard Apparatus) and a 10-μl Hamilton syringe connected via a PE-50 polyethylene catheter to the injector cannula.

### Osmotic pumps

Alzet micro-osmotic pumps (model 1002) were loaded 4 h before surgery with either vehicle saline solution (NaCl) or uncarboxylated OCN (30 ng h^−1^). Mice were anesthetized with isoflurane, and osmotic pumps were surgically installed subcutaneously in the backs of the mice. The incision in the back of the mice was sutured, and the mouse was observed in a recovery chamber until it was fully mobile. Behavioral analyses started 1 week after osmotic pump installation.

### Pharmacological modulation of autophagy

We performed intra-hippocampal injections of either 1 μg of TAT-scramble (dissolved in PBS) or 1 μg of TAT-Beclin-1 (dissolved in PBS). The TAT-Beclin-1 (YGRKKRRQRRRGGTNVFNATFEIWHDGEFGT) consisted of 11 amino acids of the TAT protein transduction domain (PTD) at the N-terminus, a GG linker to increase flexibility and 18 amino acids derived from Beclin-1—amino acids 267–284 containing three substitutions: H275E, S279D and Q281E. Control peptide, TAT-scramble (YGRKKRRQRRRGGVGNDFFINHETTGFATEW), consisted of the TAT PTD, a GG linker and a scrambled version of the C-terminal 18 amino acids from Tat-Beclin-1. All drugs were infused in a volume of 1 μl (bilaterally) in the dorsal hippocampus chronically using hippocampal cannulas for five consecutive days before behavioral tasks and maintained during the behavioral days (total, 10 d). For brain collection, hippocampal injections of TAT-scramble or TAT-Beclin-1 through cannulas were performed 12 h before euthanization.

### GPR158 mutagenesis and lentivirus constructs

Five putative ciliary localization sequences (VxPx) were identified on the C-terminal region of GPR158, at positions 212, 277, 300, 331 and 377, and mutated as follows: VCPW (212–215), (377–380) and (277–280) into ACAW; VLPG (300–303) into ALQG; and VAPK (331–334) into AAAK. The mutated GPR158 and the WT GPR158 fragments (purchased from Eurofins Genomics) were fused with the turbo EGFP and cloned into the pSicoR plasmid (by substitution of the original GFP). Lentiviral particles were produced for each construct (viral titer of 6.30 × 10^8^ TU ml^−1^ or 7.60 × 10^8^ TU ml^−1^ for the WT and mutated forms, respectively). Primary hippocampal neurons derived from Gpr158 knockout (KO) mice were infected (multiplicity of infection 10) at DIV1 with lentiviruses expressing either WT or mutated GPR158 and analyzed at DIV18 upon OCN treatment.

### AAVs expressing shRNA

All AAV9s expressing shRNA were purchased from Vector Biosystems. shRNAs specific to *Ift20* (AAV9-GFP-U6-m-*Ift20*-shRNA) (shRNA sequence (VBLKO-248329)):

5′-CCGGGGAGGAGTGCAAGGACTTTGTCTCGAGACAAAGTCCTTGCACTCCTCCTT TTT-3′, *Kif3a* (AAV9-GFP-U6-m-*Kif3a*-shRNA) (shRNA sequence (TRCN0000339511)): 5′-CCGGGGCCTGATGTGGGAGTATATACTCGAGTATATACTCCCACATCAGGCCTTT TT-3′ or scrambled non-targeting negative control (AAV9-GFP-U6-scrmb-shRNA) were bilaterally injected in a volume of 1 μl, 3 weeks before behavioral tests or brain tissue collection. The AAV titers were between 1.0 × 10^13^ GC ml^−1^ and 4.3 × 10^13^ GC ml^−1^. AAVs expressing shRNAmir target *IFT20* (AAV9-eSYN-GFP-*Ift20*-shRNAmir) with the following targeting sequence: GGAGGAGTGCAAGGACTTTGT or IFT88 (AAV9-eSYN-GFP-Ift88-shRNAmir) with the targeting sequence: GCAGGAAGACTGAAAGTGAAT. These shRNAmir sequences are in the 3′ untranslated region (UTR) of eGFP under the eSYN promoter, specifically active in neurons. The eSYN promoter is a hybrid promoter consisting of a 0.4-kb cytomegalovirus enhancer and the 0.45-kb human Synapsin 1 promoter fragment (hSYN1). AAV9-eSYN-GFP-shRNA155mir was used as control. The AAV titers were between 1.3 × 10^13^ GC ml^−1^ and 6.2 × 10^13^ GC ml^−1^. AAV overexpressing *Ift20* (AAV9-CMV-*mIft20*-IRES-eGFP) and AAV9-CMV-eGFP, used as control, had titers between 5.0 × 10^12^ GC ml^−1^ and 6.0 × 10^12^ GC ml^−1^.

### Behavioral tests

For all behavioral assessments, mice were handled for at least 20 min over three consecutive days. Before the behavioral procedure, animals were transported a short distance from the holding mouse facility to the testing room in their home cages and left undisturbed for at least 1 h before testing.

#### LDT

Each animal was placed in a 43 × 43-cm open field also containing a dark compartment and tested for 10 min. Mice were placed individually into the dark chamber of the open-field arena and allowed to explore freely. Mice were monitored throughout each test session by infrared light beam activity monitor using actiMot2 software (TSE Systems, PhenoMaster Software). Anxiety was quantified by measuring the number of entries in the light compartment, the latency to reach the light compartment and the percentage of distance spent in the light versus dark chamber of the open-field box.

NOR paradigm, three foot shock CFC, MWM and OFTs were performed and analyzed exactly as described by Glatigny et al.^20^.

### Statistics and reproducibility

All values are expressed as mean ± s.e.m. Alpha was set to 0.05 for all analyses. Statistical parameters, including the exact sample size (*n*), post hoc tests and statistical significance, are reported in every figure and figure legend. All micrographs or blots are representative of at least three replicates.

The number of mice was estimated to be sufficient on the basis of pilot experiments. Data distribution was assumed to be normal, but this was not formally tested. Data were estimated to be statistically significant when *P* ≤ 0.05 by Student’s *t*-tests and one-way or two-way ANOVAs with repeated measures when appropriate. Significant ANOVAs were followed by a two-way repeated-measures ANOVA with a post hoc Tukey’s honestly significant difference (HSD) multiple comparison test for pairwise differences between each mouse group. Data were analyzed using GraphPad Prism version 9 software. For each analyzed animal, the injection site was verified by immunohistochemistry or by qRT–PCR to check the downregulation of the targeted gene. In the case that these verifications were not correct (not injected in the hippocampus or downregulation <10%), animals were excluded.

Group sizes were determined after performing a power calculation to lead to an 80% chance of detecting a significant difference (*P* ≤ 0.05). To avoid cage effect, mice were allocated to different cages using simple randomization.

Behavioral experiments were carried out in a blinded fashion. Investigators were blinded during experimental procedures, data collection and data analysis by assigning codes (prepared by other investigators irrelevant to this study) to mice, mouse cages, cell samples and images before processing, to ensure unbiased analysis.

### Reporting summary

Further information on research design is available in the Nature Portfolio Reporting Summary linked to this article.

## Supplementary information

Reporting Summary

## Source data

Source Data Fig. 1 Statistical source data.

Source Data Fig. 2 Statistical source data.

Source Data Fig. 3 Statistical source data.

Source Data Fig. 4 Statistical source data.

Source Data Fig. 5 Statistical source data.

Source Data Fig. 6 Statistical source data.

Source Data Fig. 7 Statistical source data.

Source Data Unprocessed western blots.

Source Data Extended Data Fig. 1 Statistical source data.

Source Data Extended Data Fig. 2 Statistical source data.

Source Data Extended Data Fig. 3 Statistical source data.

Source Data Extended Data Fig. 4 Statistical source data.

Source Data Extended Data Fig. 5 Statistical source data.

Source Data Extended Data Fig. 6 Statistical source data.

Source Data Extended Data Fig. 7 Statistical source data.

Source Data Extended Data Unprocessed western blots.

## Data Availability

Raw data of the proteomic analysis have been deposited in PRIDE under accession code PXD056975. All other data are available from the corresponding author upon reasonable request.

## Code availability

The code used for the analysis of the proteomic data is available on Dryad at 10.5061/dryad.dfn2z35bh.

## References

[1] Lopez-Otin, C., Blasco, M. A., Partridge, L., Serrano, M. & Kroemer, G. Hallmarks of aging: an expanding universe. *Cell* **186**, 243–278 (2023). 36599349 10.1016/j.cell.2022.11.001

[2] Kramer, A. F. et al. Ageing, fitness and neurocognitive function. *Nature* **400**, 418–419 (1999). 10440369 10.1038/22682

[3] Bishop, N. A., Lu, T. & Yankner, B. A. Neural mechanisms of ageing and cognitive decline. *Nature* **464**, 529–535 (2010). 20336135 10.1038/nature08983 PMC2927852

[4] Fan, X., Wheatley, E. G. & Villeda, S. A. Mechanisms of hippocampal aging and the potential for rejuvenation. *Annu. Rev. Neurosci.* **40**, 251–272 (2017). 28441118 10.1146/annurev-neuro-072116-031357

[5] Bartsch, T. & Wulff, P. The hippocampus in aging and disease: from plasticity to vulnerability. *Neuroscience* **309**, 1–16 (2015). 26241337 10.1016/j.neuroscience.2015.07.084

[6] Wheatley, E. G. et al. Neuronal O-GlcNAcylation improves cognitive function in the aged mouse brain. *Curr. Biol.* **29**, 3359–3369(2019). 31588002 10.1016/j.cub.2019.08.003 PMC7199460

[7] Leuner, B. & Gould, E. Dendritic growth in medial prefrontal cortex and cognitive flexibility are enhanced during the postpartum period. *J. Neurosci.* **30**, 13499–13503 (2010). 20926675 10.1523/JNEUROSCI.3388-10.2010 PMC2963448

[8] Mattson, M. P. & Magnus, T. Ageing and neuronal vulnerability. *Nat. Rev. Neurosci.* **7**, 278–294 (2006). 16552414 10.1038/nrn1886 PMC3710114

[9] Bieri, G., Schroer, A. B. & Villeda, S. A. Blood-to-brain communication in aging and rejuvenation. *Nat. Neurosci.* **26**, 379–393 (2023). 36646876 10.1038/s41593-022-01238-8

[10] Lehallier, B. et al. Undulating changes in human plasma proteome profiles across the lifespan. *Nat. Med.* **25**, 1843–1850 (2019). 31806903 10.1038/s41591-019-0673-2 PMC7062043

[11] Schaum, N. et al. Ageing hallmarks exhibit organ-specific temporal signatures. *Nature* **583**, 596–602 (2020). 32669715 10.1038/s41586-020-2499-y PMC7757734

[12] Villeda, S. A. et al. Young blood reverses age-related impairments in cognitive function and synaptic plasticity in mice. *Nat. Med.* **20**, 659–663 (2014). 24793238 10.1038/nm.3569 PMC4224436

[13] Villeda, S. A. et al. The ageing systemic milieu negatively regulates neurogenesis and cognitive function. *Nature* **477**, 90–94 (2011). 21886162 10.1038/nature10357 PMC3170097

[14] Castellano, J. M. et al. Human umbilical cord plasma proteins revitalize hippocampal function in aged mice. *Nature* **544**, 488–492 (2017). 28424512 10.1038/nature22067 PMC5586222

[15] Wyss-Coray, T. Ageing, neurodegeneration and brain rejuvenation. *Nature* **539**, 180–186 (2016). 27830812 10.1038/nature20411 PMC5172605

[16] Horowitz, A. M. et al. Blood factors transfer beneficial effects of exercise on neurogenesis and cognition to the aged brain. *Science* **369**, 167–173 (2020). 32646997 10.1126/science.aaw2622 PMC7879650

[17] Oury, F. et al. Maternal and offspring pools of osteocalcin influence brain development and functions. *Cell* **155**, 228–241 (2013). 24074871 10.1016/j.cell.2013.08.042 PMC3864001

[18] Khrimian, L. et al. Gpr158 mediates osteocalcin’s regulation of cognition. *J. Exp. Med.* **214**, 2859–2873 (2017). 28851741 10.1084/jem.20171320 PMC5626410

[19] Kosmidis, S. et al. RbAp48 protein is a critical component of GPR158/OCN signaling and ameliorates age-related memory loss. *Cell Rep.* **25**, 959–973 (2018). 30355501 10.1016/j.celrep.2018.09.077 PMC7725275

[20] Glatigny, M. et al. Autophagy is required for memory formation and reverses age-related memory decline. *Curr. Biol.* **29**, 435–448 (2019). 30661803 10.1016/j.cub.2018.12.021

[21] Lamb, C. A., Yoshimori, T. & Tooze, S. A. The autophagosome: origins unknown, biogenesis complex. *Nat. Rev. Mol. Cell Biol.* **14**, 759–774 (2013). 24201109 10.1038/nrm3696

[22] Mizushima, N. & Komatsu, M. Autophagy: renovation of cells and tissues. *Cell* **147**, 728–741 (2011). 22078875 10.1016/j.cell.2011.10.026

[23] Ktistakis, N. T. & Tooze, S. A. Digesting the expanding mechanisms of autophagy. *Trends Cell Biol.* **26**, 624–635 (2016). 27050762 10.1016/j.tcb.2016.03.006

[24] Kulkarni, A., Chen, J. & Maday, S. Neuronal autophagy and intercellular regulation of homeostasis in the brain. *Curr. Opin. Neurobiol.* **51**, 29–36 (2018). 29529415 10.1016/j.conb.2018.02.008

[25] Fleming, A. et al. The different autophagy degradation pathways and neurodegeneration. *Neuron* **110**, 935–966 (2022). 35134347 10.1016/j.neuron.2022.01.017 PMC8930707

[26] Aman, Y. et al. Autophagy in healthy aging and disease. *Nat. Aging* **1**, 634–650 (2021). 34901876 10.1038/s43587-021-00098-4 PMC8659158

[27] Klionsky, D. J. et al. Autophagy in major human diseases. *EMBO J.* **40**, e108863 (2021). 34459017 10.15252/embj.2021108863 PMC8488577

[28] Levine, B. & Kroemer, G. Biological functions of autophagy genes: a disease perspective. *Cell* **176**, 11–42 (2019). 30633901 10.1016/j.cell.2018.09.048 PMC6347410

[29] Galluzzi, L., Pietrocola, F., Levine, B. & Kroemer, G. Metabolic control of autophagy. *Cell* **159**, 1263–1276 (2014). 25480292 10.1016/j.cell.2014.11.006 PMC4500936

[30] Pandey, K., Yu, X. W., Steinmetz, A. & Alberini, C. M. Autophagy coupled to translation is required for long-term memory. *Autophagy* **17**, 1614–1635 (2021). 32501746 10.1080/15548627.2020.1775393 PMC8354608

[31] Gupta, V. K. et al. Spermidine suppresses age-associated memory impairment by preventing adverse increase of presynaptic active zone size and release. *PLoS Biol.* **14**, e1002563 (2016). 27684064 10.1371/journal.pbio.1002563 PMC5042543

[32] Nikoletopoulou, V., Sidiropoulou, K., Kallergi, E., Dalezios, Y. & Tavernarakis, N. Modulation of autophagy by BDNF underlies synaptic plasticity. *Cell Metab.* **26**, 230–242 (2017). 28683289 10.1016/j.cmet.2017.06.005

[33] Bhukel, A. et al. Autophagy within the mushroom body protects from synapse aging in a non-cell autonomous manner. *Nat. Commun.* **10**, 1318 (2019). 30899013 10.1038/s41467-019-09262-2 PMC6428838

[34] De Risi, M. et al. Mechanisms by which autophagy regulates memory capacity in ageing. *Aging Cell* **19**, e13189 (2020). 32729663 10.1111/acel.13189 PMC7511873

[35] Moigneu, C. et al. Systemic GDF11 attenuates depression-like phenotype in aged mice via stimulation of neuronal autophagy. *Nat. Aging* **3**, 213–228 (2023). 37118117 10.1038/s43587-022-00352-3 PMC10154197

[36] Boukhalfa, A., Roccio, F., Dupont, N., Codogno, P. & Morel, E. The autophagy protein ATG16L1 cooperates with IFT20 and INPP5E to regulate the turnover of phosphoinositides at the primary cilium. *Cell Rep.* **35**, 109045 (2021). 33910006 10.1016/j.celrep.2021.109045

[37] Miceli, C. et al. The primary cilium and lipophagy translate mechanical forces to direct metabolic adaptation of kidney epithelial cells. *Nat. Cell Biol.* **22**, 1091–1102 (2020). 32868900 10.1038/s41556-020-0566-0

[38] Morleo, M. & Franco, B. The autophagy-cilia axis: an intricate relationship. *Cells* **8**, 905 (2019). 31443299 10.3390/cells8080905 PMC6721705

[39] Orhon, I., Dupont, N., Pampliega, O., Cuervo, A. M. & Codogno, P. Autophagy and regulation of cilia function and assembly. *Cell Death Differ.* **22**, 389–397 (2015). 25361082 10.1038/cdd.2014.171 PMC4326575

[40] Zemirli, N. et al. The primary cilium protein folliculin is part of the autophagy signaling pathway to regulate epithelial cell size in response to fluid flow. *Cell Stress* **3**, 100–109 (2019). 31225504 10.15698/cst2019.03.180 PMC6551741

[41] Valente, E. M., Rosti, R. O., Gibbs, E. & Gleeson, J. G. Primary cilia in neurodevelopmental disorders. *Nat. Rev. Neurol.* **10**, 27–36 (2014). 24296655 10.1038/nrneurol.2013.247 PMC3989897

[42] Rhee, S., Kirschen, G. W., Gu, Y. & Ge, S. Depletion of primary cilia from mature dentate granule cells impairs hippocampus-dependent contextual memory. *Sci. Rep.* **6**, 34370 (2016). 27678193 10.1038/srep34370 PMC5039642

[43] Berbari, N. F. et al. Hippocampal and cortical primary cilia are required for aversive memory in mice. *PLoS ONE* **9**, e106576 (2014). 25184295 10.1371/journal.pone.0106576 PMC4153651

[44] Wang, Z., Phan, T. & Storm, D. R. The type 3 adenylyl cyclase is required for novel object learning and extinction of contextual memory: role of cAMP signaling in primary cilia. *J. Neurosci.* **31**, 5557–5561 (2011). 21490195 10.1523/JNEUROSCI.6561-10.2011 PMC3091825

[45] Marinissen, M. J. & Gutkind, J. S. G-protein-coupled receptors and signaling networks: emerging paradigms. *Trends Pharmacol. Sci.* **22**, 368–376 (2001). 11431032 10.1016/s0165-6147(00)01678-3

[46] Hilgendorf, K. I., Johnson, C. T. & Jackson, P. K. The primary cilium as a cellular receiver: organizing ciliary GPCR signaling. *Curr. Opin. Cell Biol.* **39**, 84–92 (2016). 26926036 10.1016/j.ceb.2016.02.008 PMC4828300

[47] Insinna, C. & Besharse, J. C. Intraflagellar transport and the sensory outer segment of vertebrate photoreceptors. *Dev. Dyn.* **237**, 1982–1992 (2008). 18489002 10.1002/dvdy.21554 PMC2692564

[48] Green, J. A. et al. Recruitment of β-arrestin into neuronal cilia modulates somatostatin receptor subtype 3 ciliary localization. *Mol. Cell. Biol.* **36**, 223–235 (2016). 26503786 10.1128/MCB.00765-15 PMC4702597

[49] Mykytyn, K. & Askwith, C. G-protein-coupled receptor signaling in cilia. *Cold Spring Harb. Perspect. Biol.* **9**, a028183 (2017). 28159877 10.1101/cshperspect.a028183 PMC5585845

[50] Ishikawa, H. & Marshall, W. F. Intraflagellar transport and ciliary dynamics. *Cold Spring Harb. Perspect. Biol.* **9**, a021998 (2017). 28249960 10.1101/cshperspect.a021998 PMC5334256

[51] Yang, D. J., Hong, J. & Kim, K. W. Hypothalamic primary cilium: a hub for metabolic homeostasis. *Exp. Mol. Med.* **53**, 1109–1115 (2021). 34211092 10.1038/s12276-021-00644-5 PMC8333261

[52] Mukhopadhyay, S. et al. TULP3 bridges the IFT-A complex and membrane phosphoinositides to promote trafficking of G protein-coupled receptors into primary cilia. *Genes Dev.* **24**, 2180–2193 (2010). 20889716 10.1101/gad.1966210 PMC2947770

[53] Fioriti, L. et al. The persistence of hippocampal-based memory requires protein synthesis mediated by the prion-like protein CPEB3. *Neuron* **86**, 1433–1448 (2015). 26074003 10.1016/j.neuron.2015.05.021

[54] Vorhees, C. V. & Williams, M. T. Morris water maze: procedures for assessing spatial and related forms of learning and memory. *Nat. Protoc.* **1**, 848–858 (2006). 17406317 10.1038/nprot.2006.116 PMC2895266

[55] Shoji-Kawata, S. et al. Identification of a candidate therapeutic autophagy-inducing peptide. *Nature* **494**, 201–206 (2013). 23364696 10.1038/nature11866 PMC3788641

[56] Schimanski, L. A. & Barnes, C. A. Neural protein synthesis during aging: effects on plasticity and memory. *Front. Aging Neurosci.* **2**, 26 (2010). 20802800 10.3389/fnagi.2010.00026 PMC2928699

[57] Pugazhenthi, S., Wang, M., Pham, S., Sze, C. I. & Eckman, C. B. Downregulation of CREB expression in Alzheimer’s brain and in Aβ-treated rat hippocampal neurons. *Mol. Neurodegener.* **6**, 60 (2011). 21854604 10.1186/1750-1326-6-60 PMC3174124

[58] Paramanik, V. & Thakur, M. K. Role of CREB signaling in aging brain. *Arch. Ital. Biol.* **151**, 33–42 (2013). 23807618 10.4449/aib.v151i1.1461

[59] Shim, M. S., Nettesheim, A., Dixon, A. & Liton, P. B. Primary cilia and the reciprocal activation of AKT and SMAD2/3 regulate stretch-induced autophagy in trabecular meshwork cells. *Proc. Natl Acad. Sci. USA* **118**, e2021942118 (2021). 33753495 10.1073/pnas.2021942118 PMC8020776

[60] Carloni, S. et al. Activation of autophagy and Akt/CREB signaling play an equivalent role in the neuroprotective effect of rapamycin in neonatal hypoxia-ischemia. *Autophagy* **6**, 366–377 (2010). 20168088 10.4161/auto.6.3.11261

[61] Hu, M. et al. Autophagy and Akt/CREB signalling play an important role in the neuroprotective effect of nimodipine in a rat model of vascular dementia. *Behav. Brain Res.* **325**, 79–86 (2017). 27923588 10.1016/j.bbr.2016.11.053

[62] Qian, Z. et al. Osteocalcin attenuates oligodendrocyte differentiation and myelination via GPR37 signaling in the mouse brain. *Sci. Adv.* **7**, eabi5811 (2021). 34678058 10.1126/sciadv.abi5811 PMC8535816

[63] Wachten, D. & Mick, D. U. Signal transduction in primary cilia—analyzing and manipulating GPCR and second messenger signaling. *Pharmacol. Ther.* **224**, 107836 (2021). 33744260 10.1016/j.pharmthera.2021.107836

[64] Guadiana, S. M. et al. Type 3 adenylyl cyclase and somatostatin receptor 3 expression persists in aged rat neocortical and hippocampal neuronal cilia. *Front. Aging Neurosci.* **8**, 127 (2016). 27303293 10.3389/fnagi.2016.00127 PMC4885836

[65] Wang, W. et al. Intraflagellar transport proteins as regulators of primary cilia length. *Front. Cell Dev. Biol.* **9**, 661350 (2021). 34095126 10.3389/fcell.2021.661350 PMC8170031

[66] Jovasevic, V. et al. Primary cilia are required for the persistence of memory and stabilization of perineuronal nets. *iScience* **24**, 102617 (2021). 34142063 10.1016/j.isci.2021.102617 PMC8185192

