# Supplementary figures and images for: A primary cilia–autophagy axis in hippocampal neurons is essential to maintain cognitive resilience
Source: Nat Aging. 2025 Feb 21;5(3):450–67. doi: 10.1038/s43587-024-00791-0 (PMC11922775; doi:10.1038/s43587-024-00791-0)

Fig 1D

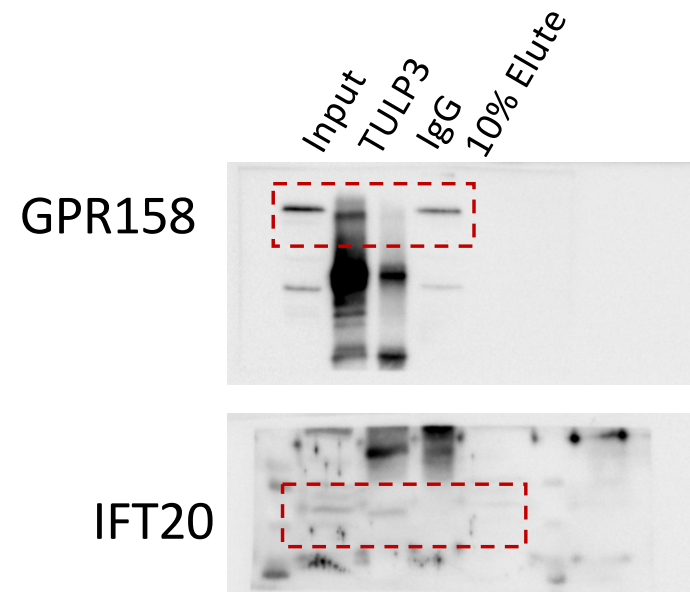

Fig 2A-1

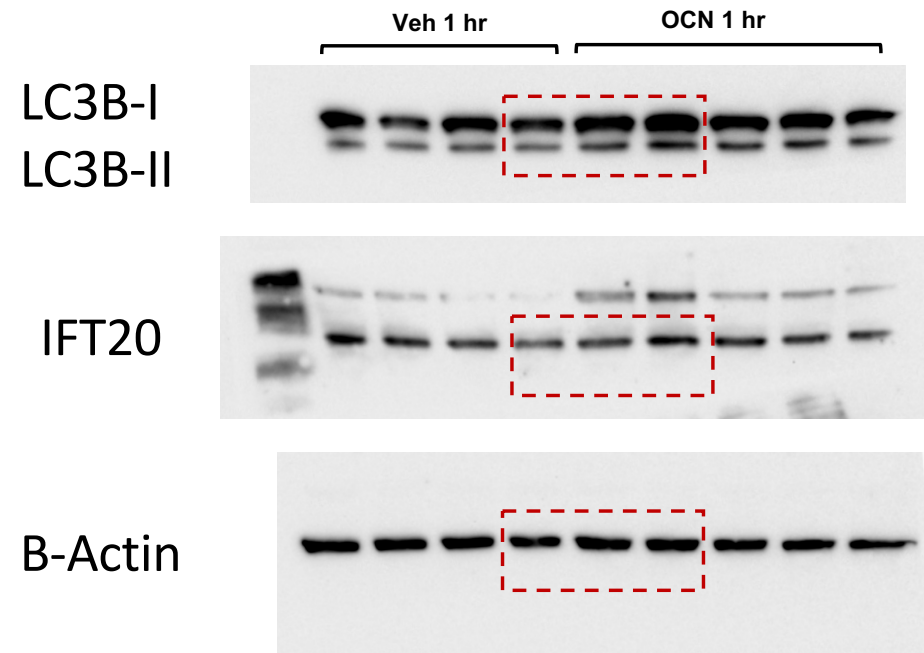

Fig 2A-2

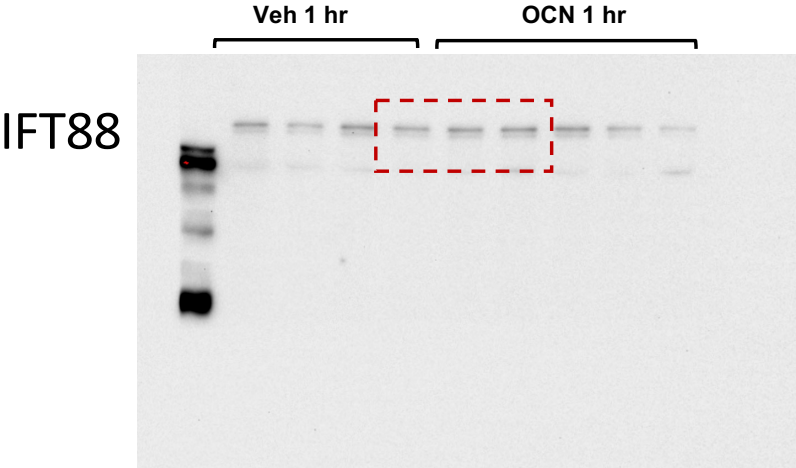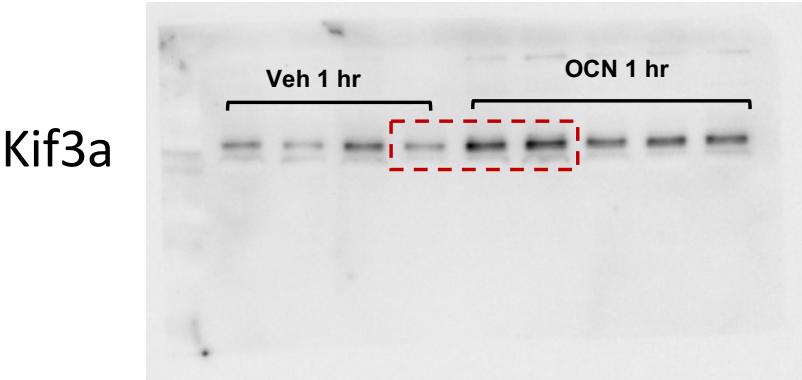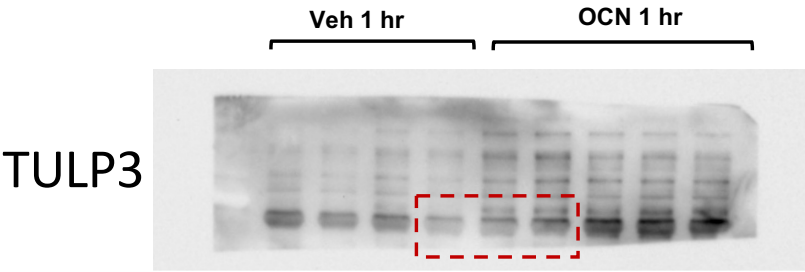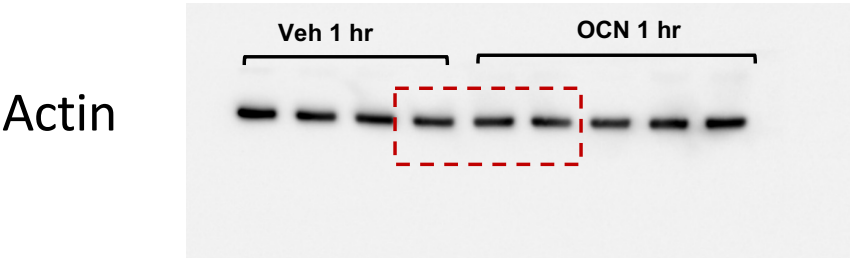

Fig 2B

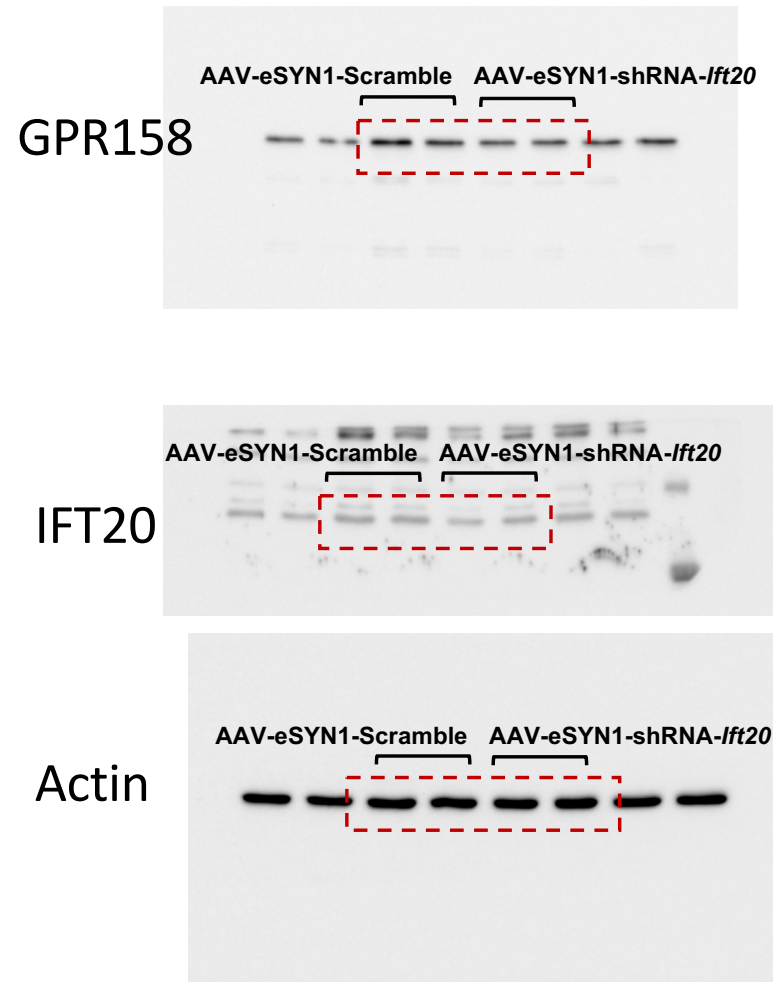

Fig 2C

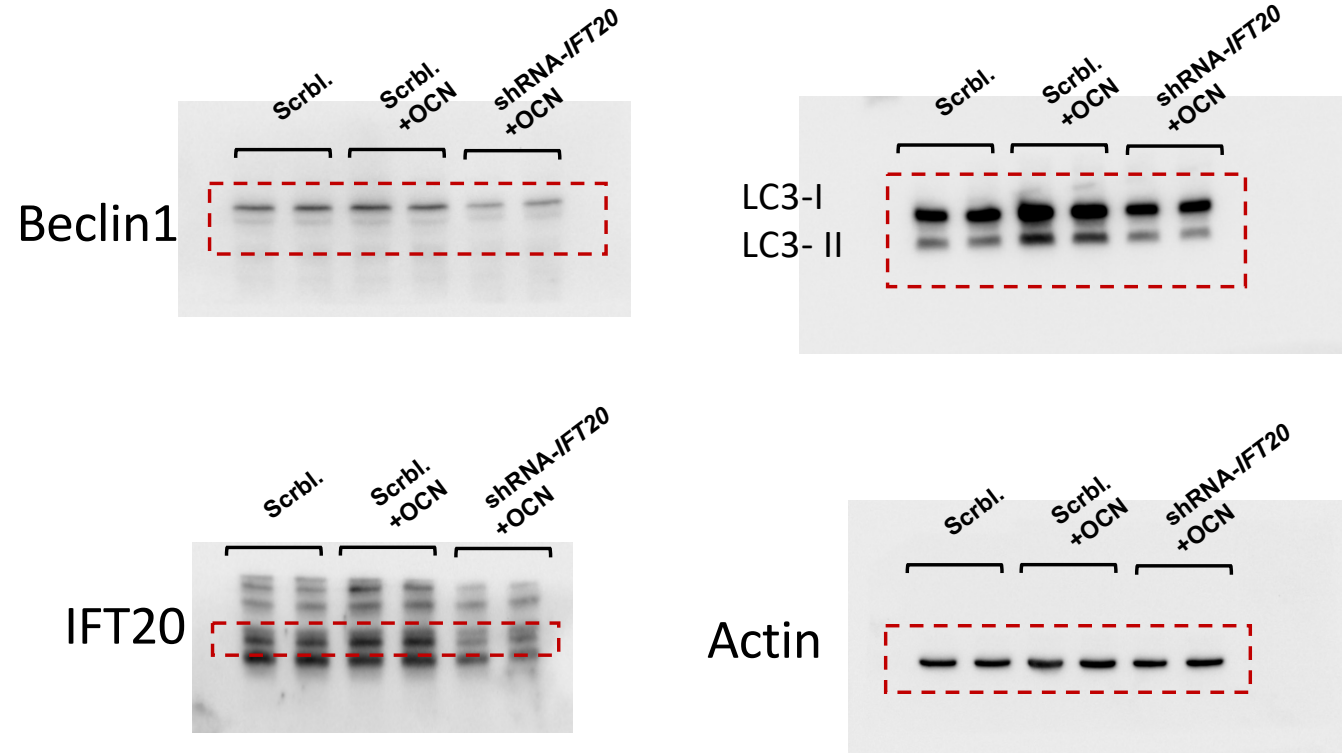

Fig 3B

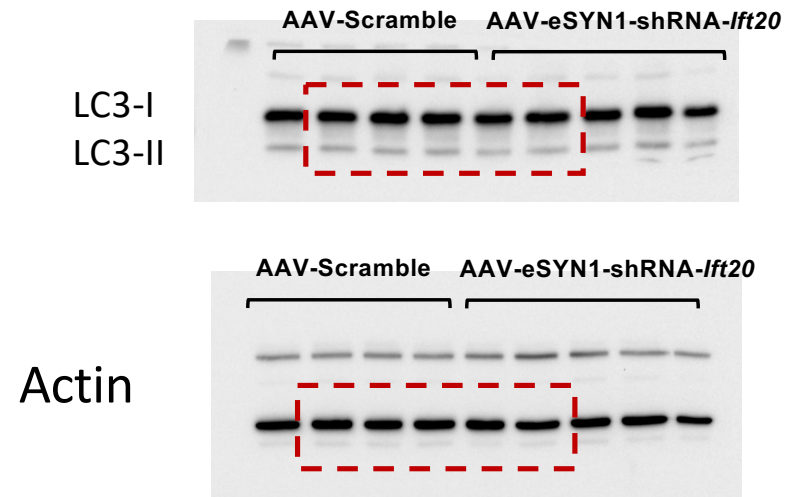

Fig 4G

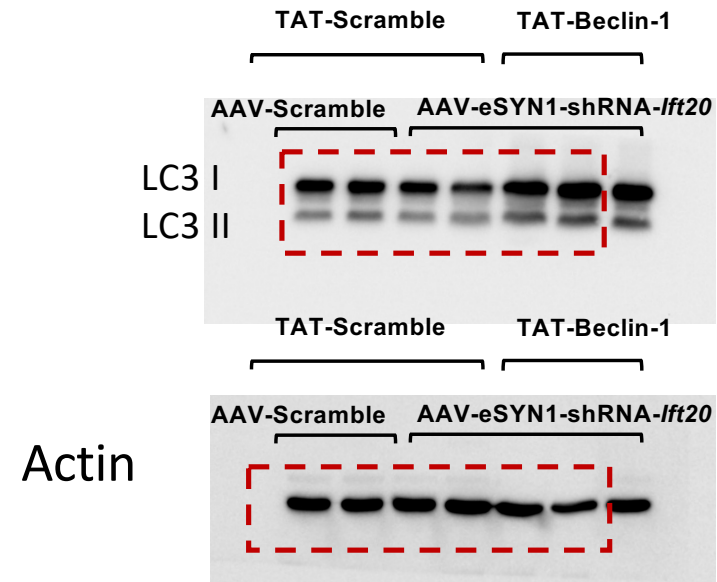

Fig 5B

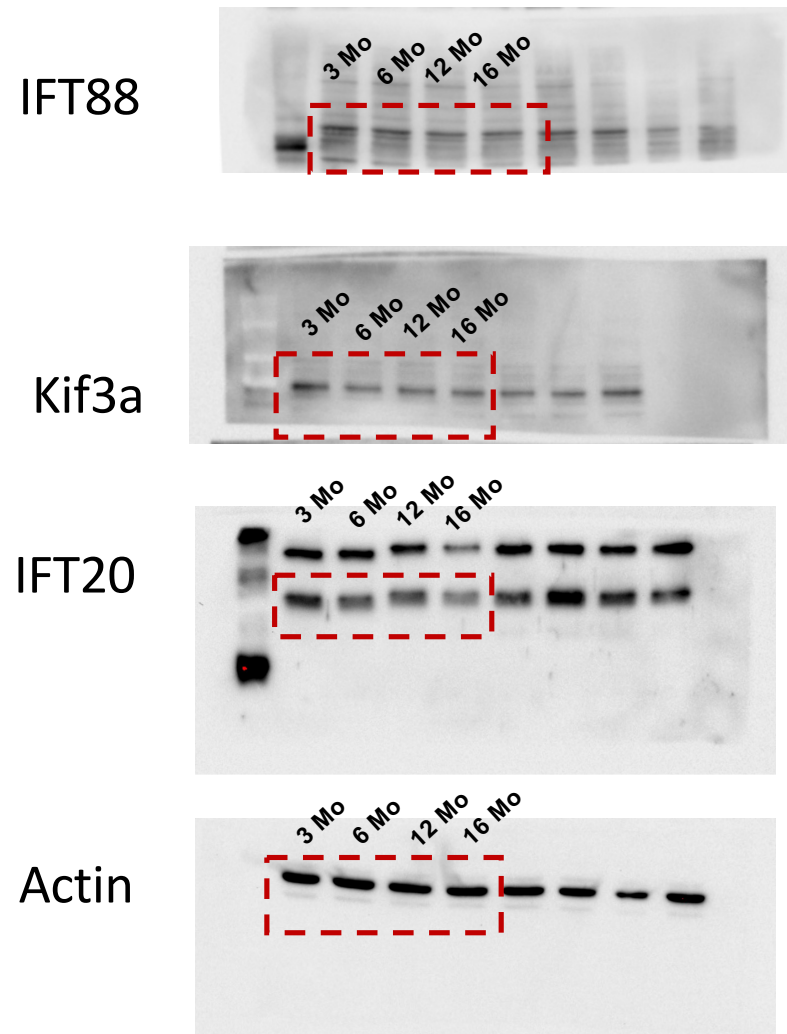

Fig 5D

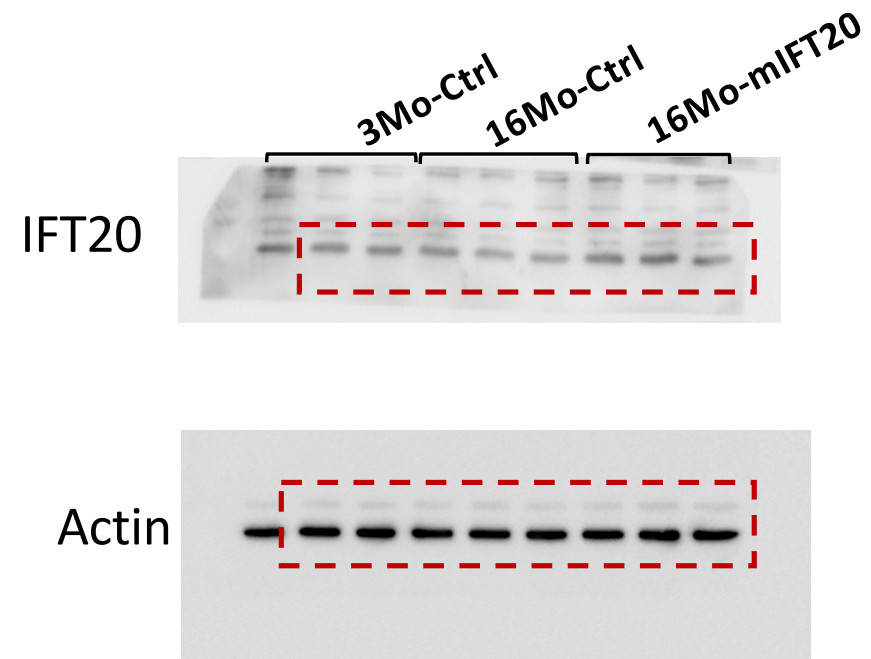

Fig 5E

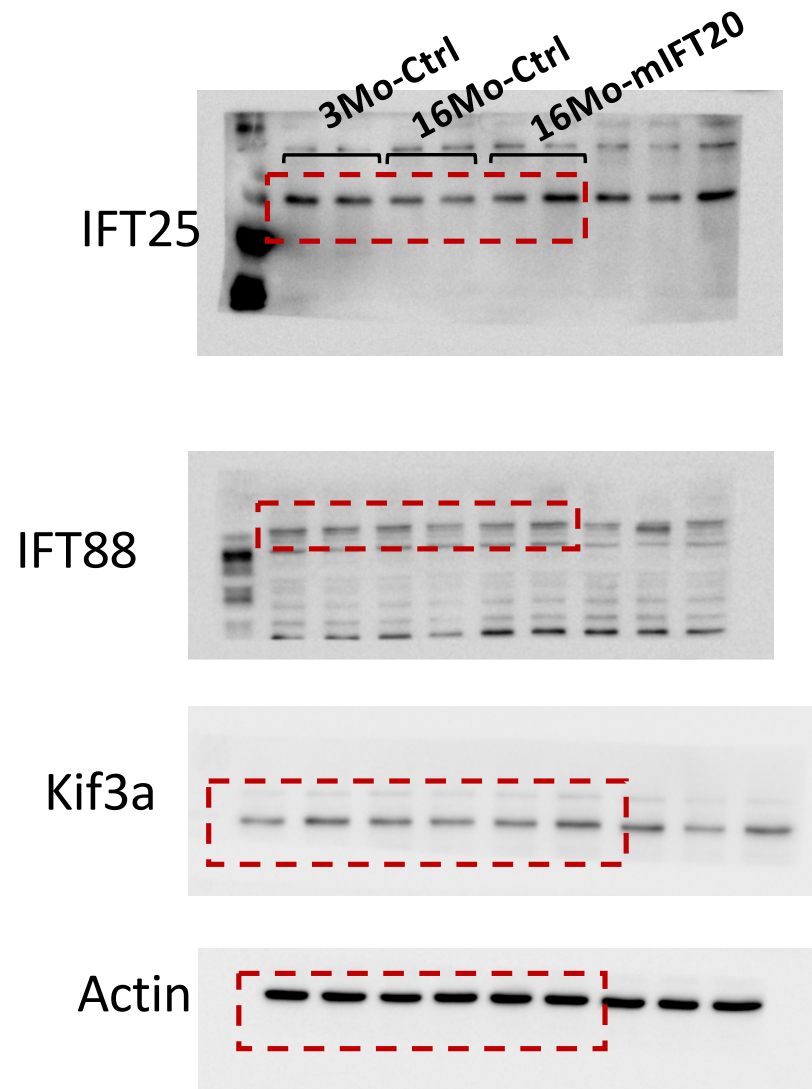

Fig 6A

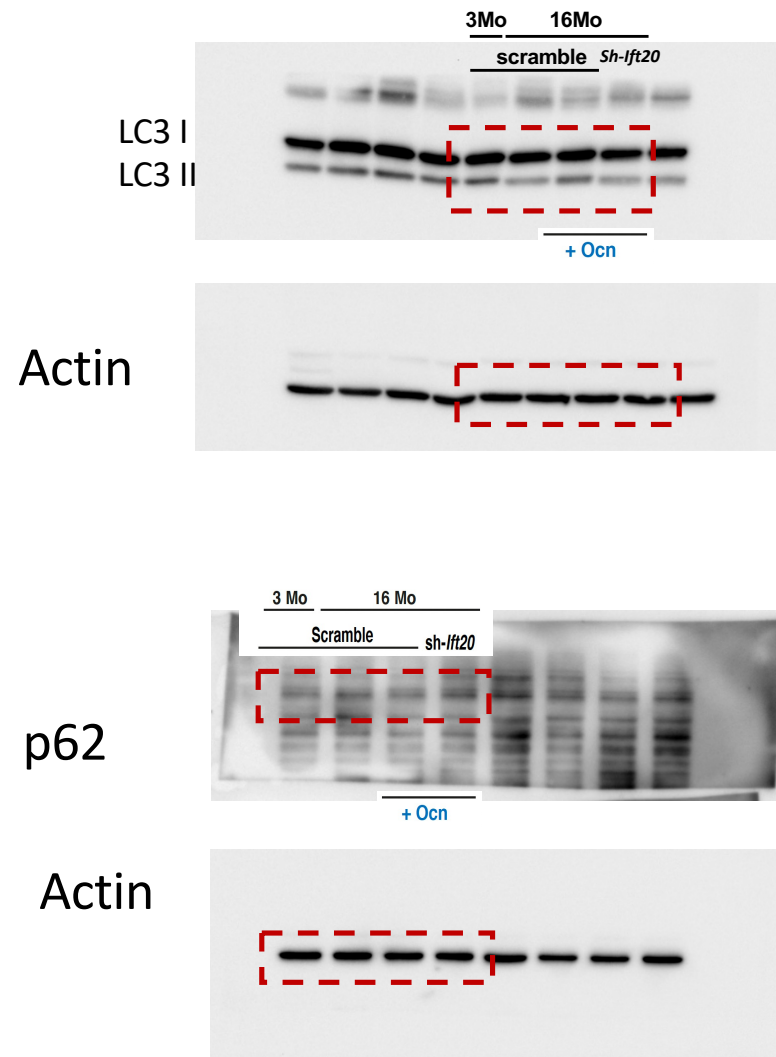

Fig 7A

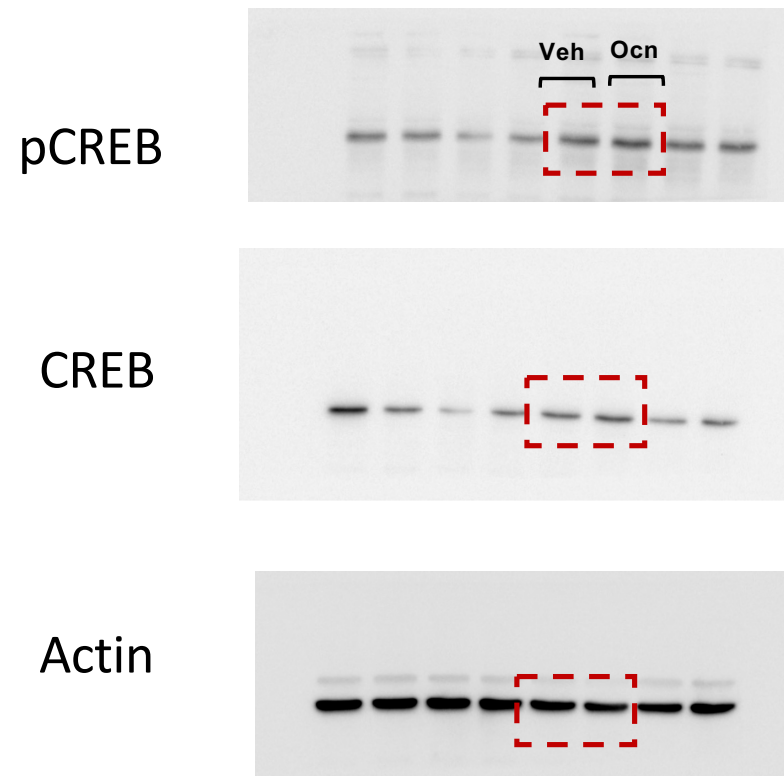

Fig 7B

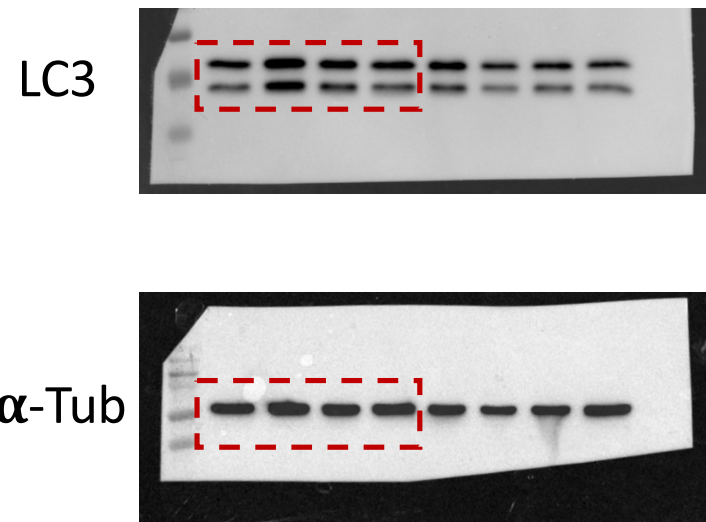

Fig 7C

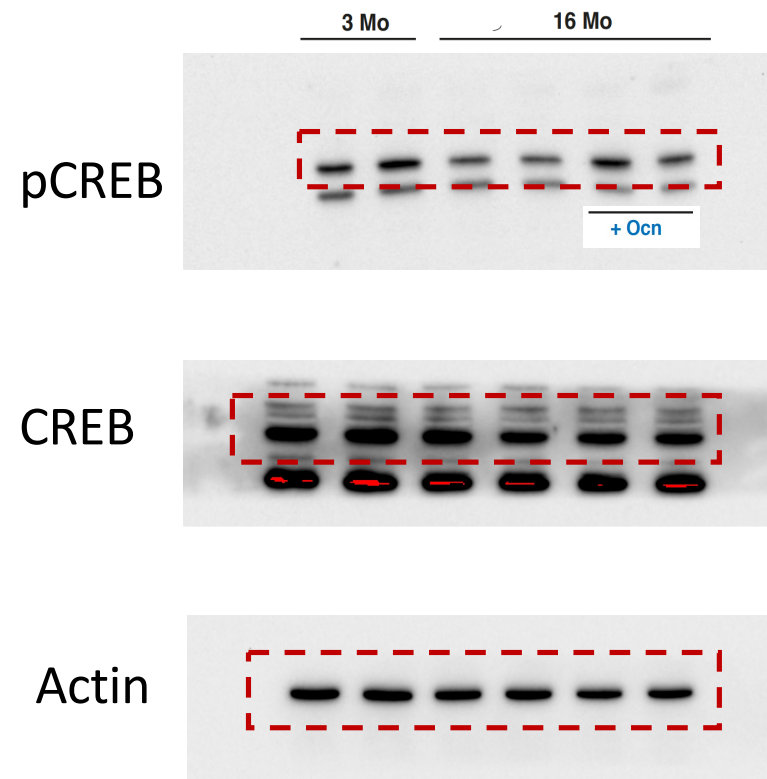

Fig 7D

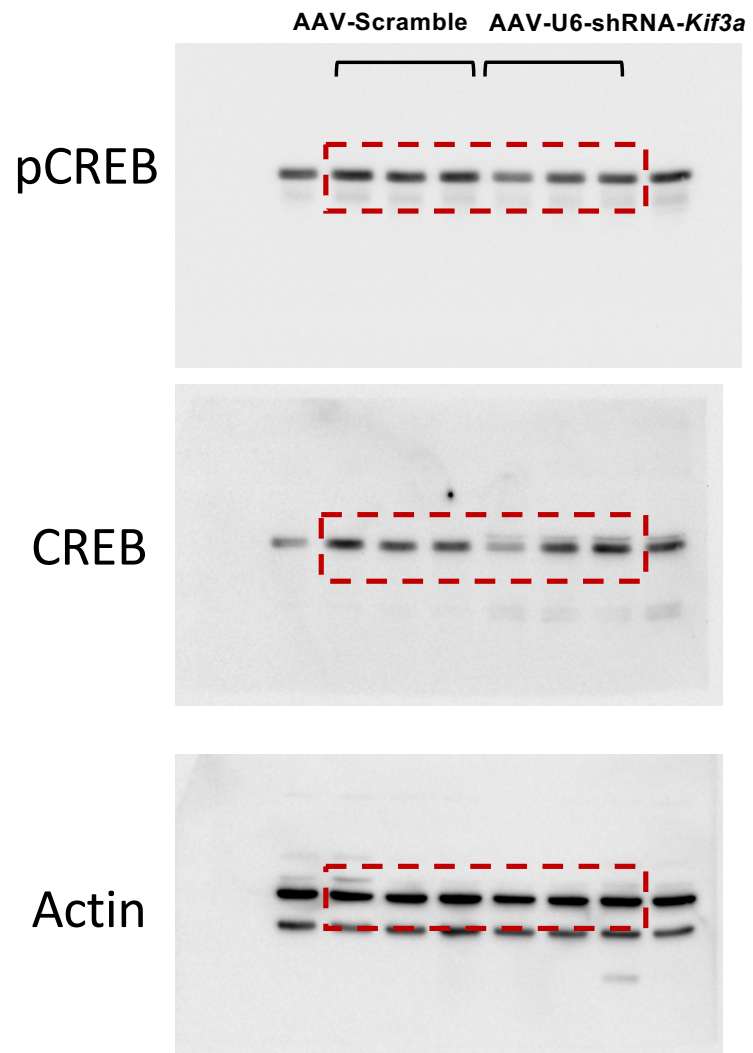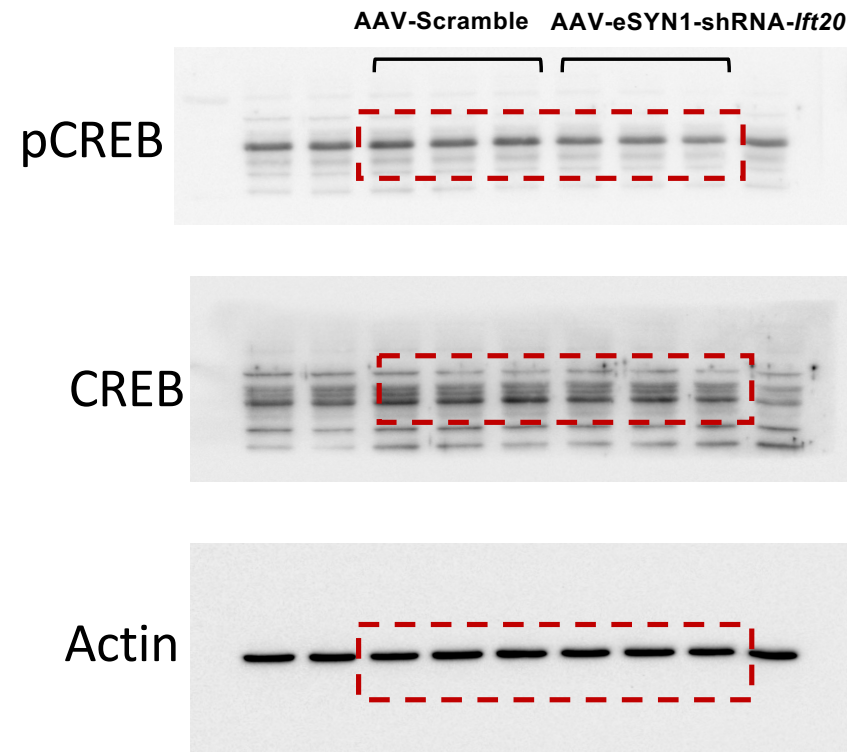

Fig 7F

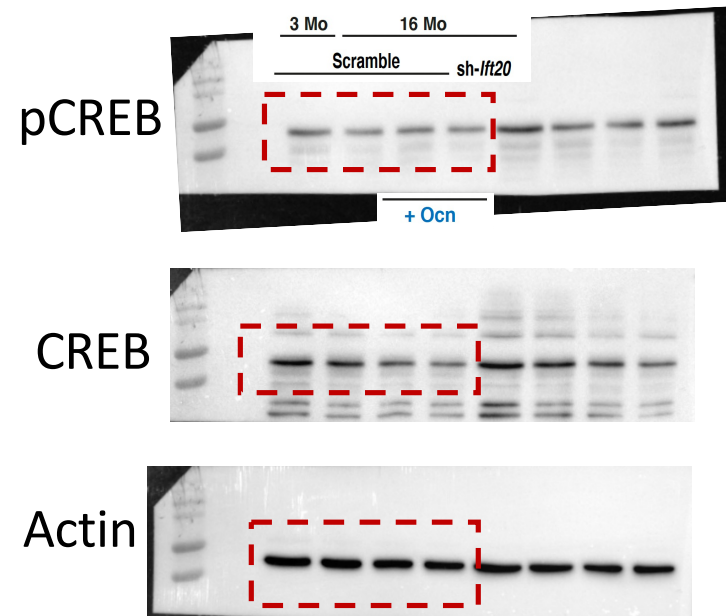

Supplement: Supplementary file 9 — Unprocessed western blots. [file 43587_2024_791_MOESM9_ESM.pdf]
