# Supplementary material for: A primary cilia–autophagy axis in hippocampal neurons is essential to maintain cognitive resilience
Source: Nat Aging. 2025 Feb 21;5(3):450–67. doi: 10.1038/s43587-024-00791-0 (PMC11922775; doi:10.1038/s43587-024-00791-0)

Extended data Fig S1D

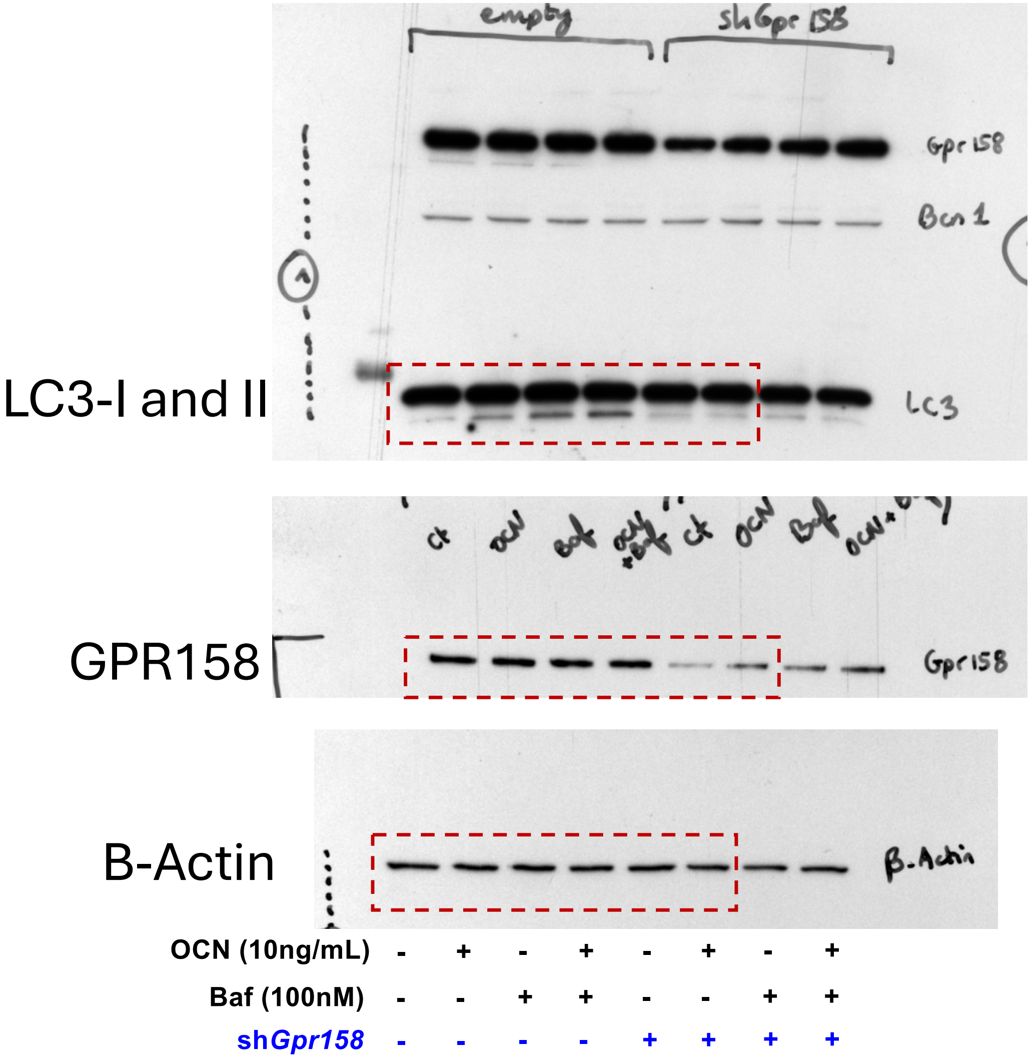

Extended data Fig S1E

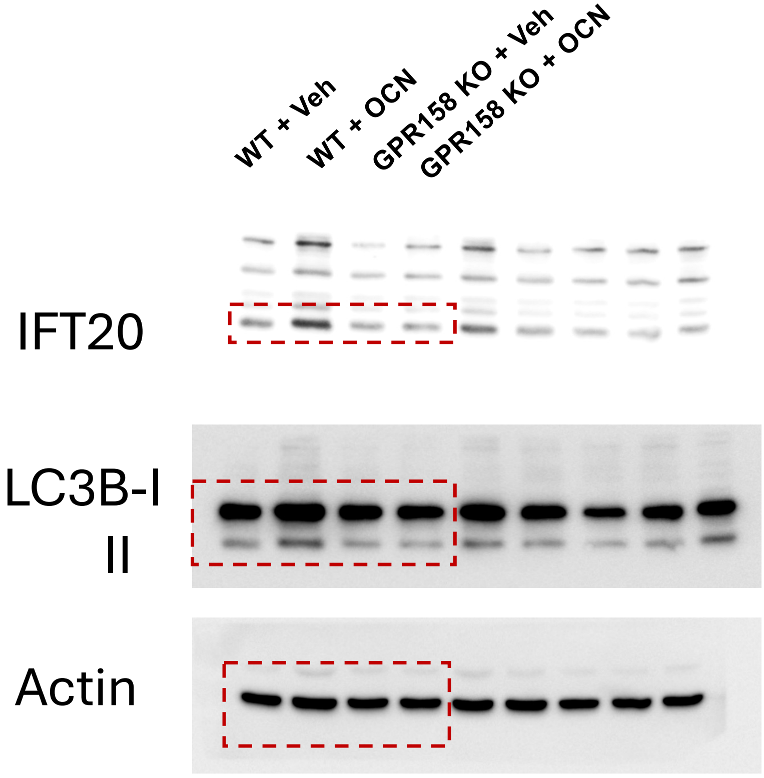

Extended data Fig S2A

IFT88

Veh 4 hr OCN 4 hr

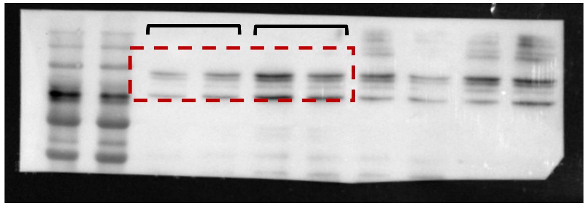

TULP3

Veh 4 hr OCN 4 hr

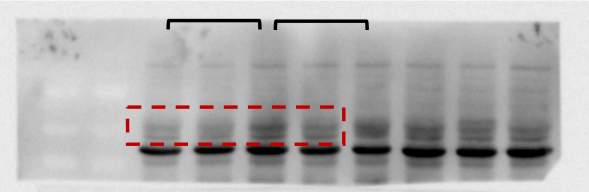

IFT25

Veh 4 hr OCN 4 hr

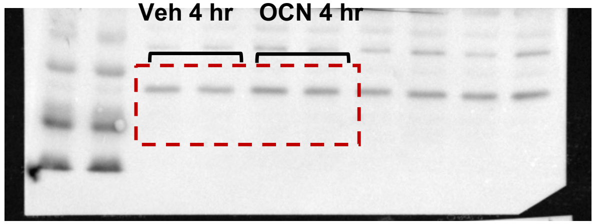

Kif3a

Veh 4 hr OCN 4 hr

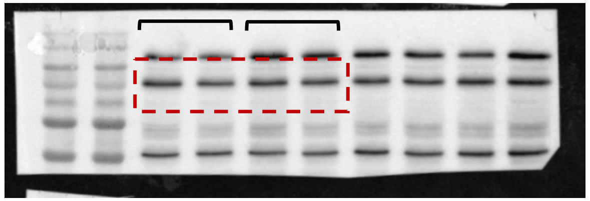

IFT20

Veh 4 hr OCN 4 hr

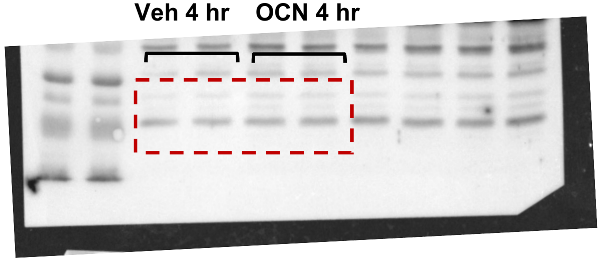

Actin

Veh 4 hr OCN 4 hr

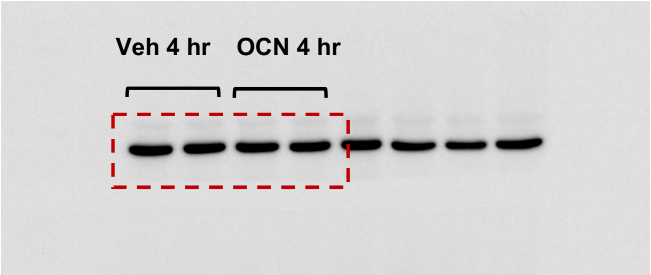

Extended data Fig S2B-1

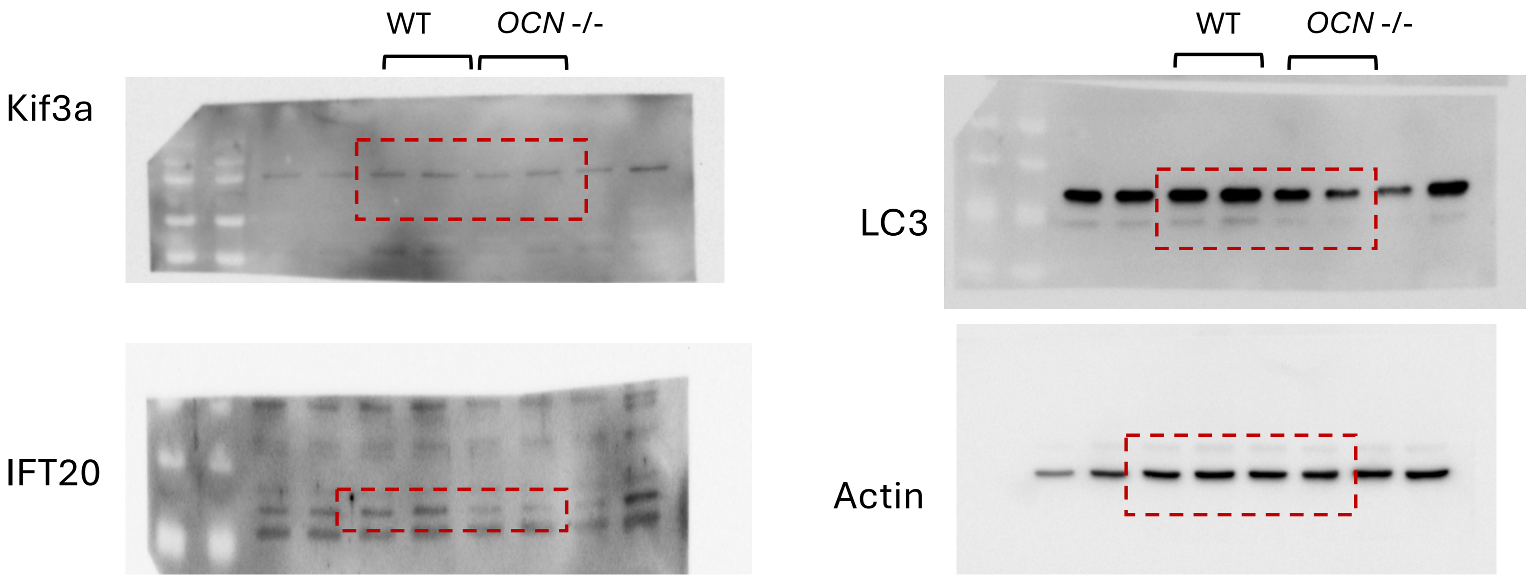

Extended data Fig S2B-2

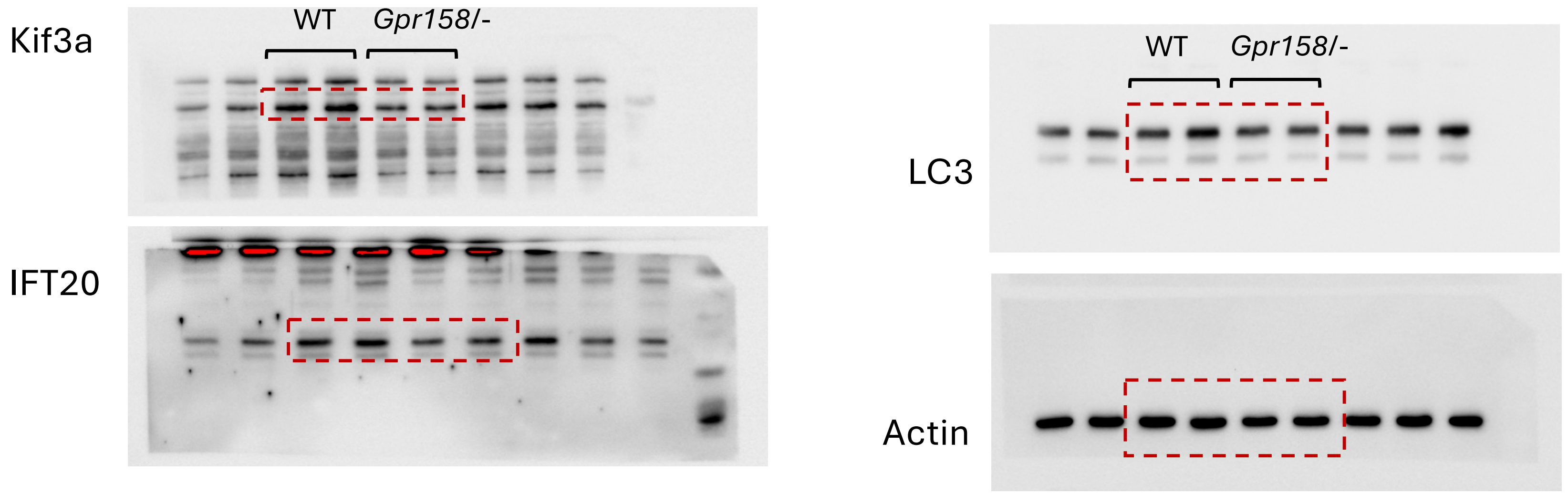

Extended data Fig S3D

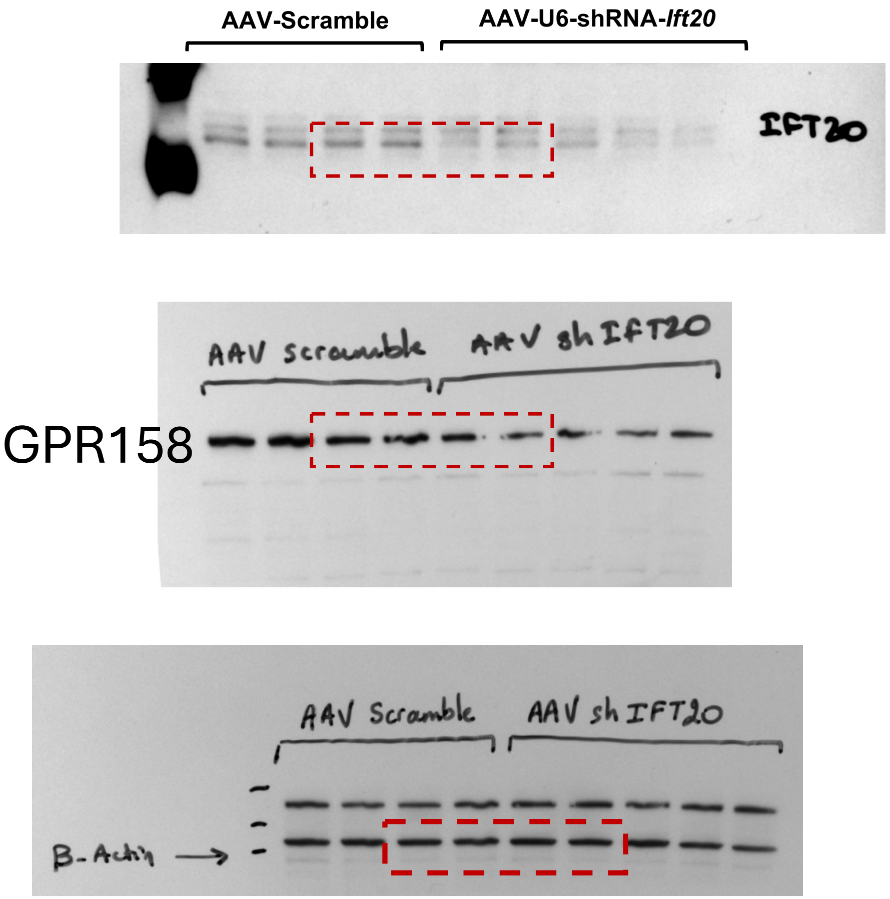

Extended data Fig S3E

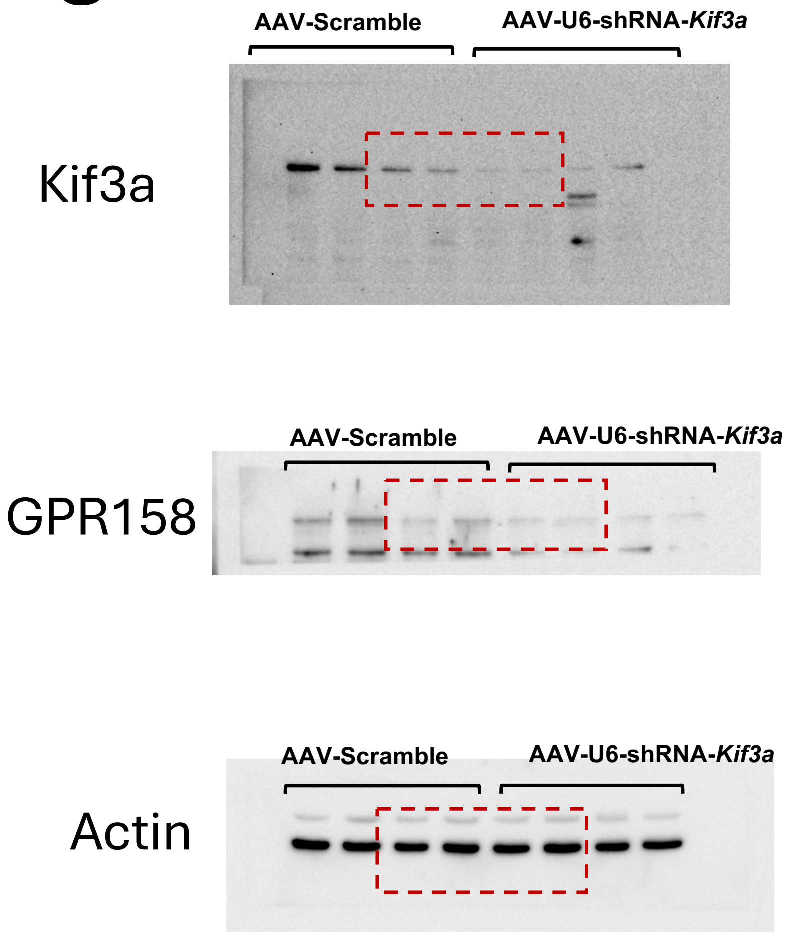

Extended data Fig S4D

IFT88

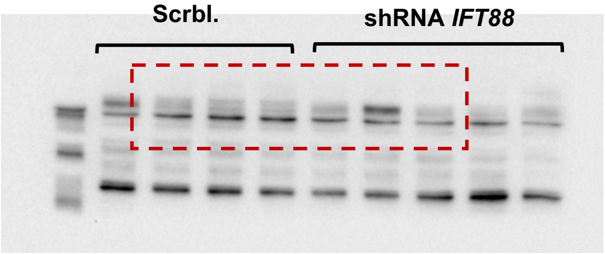

Actin

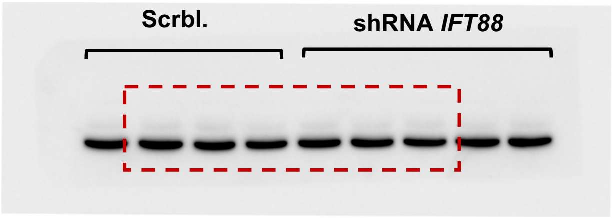

Extended data Fig S6A

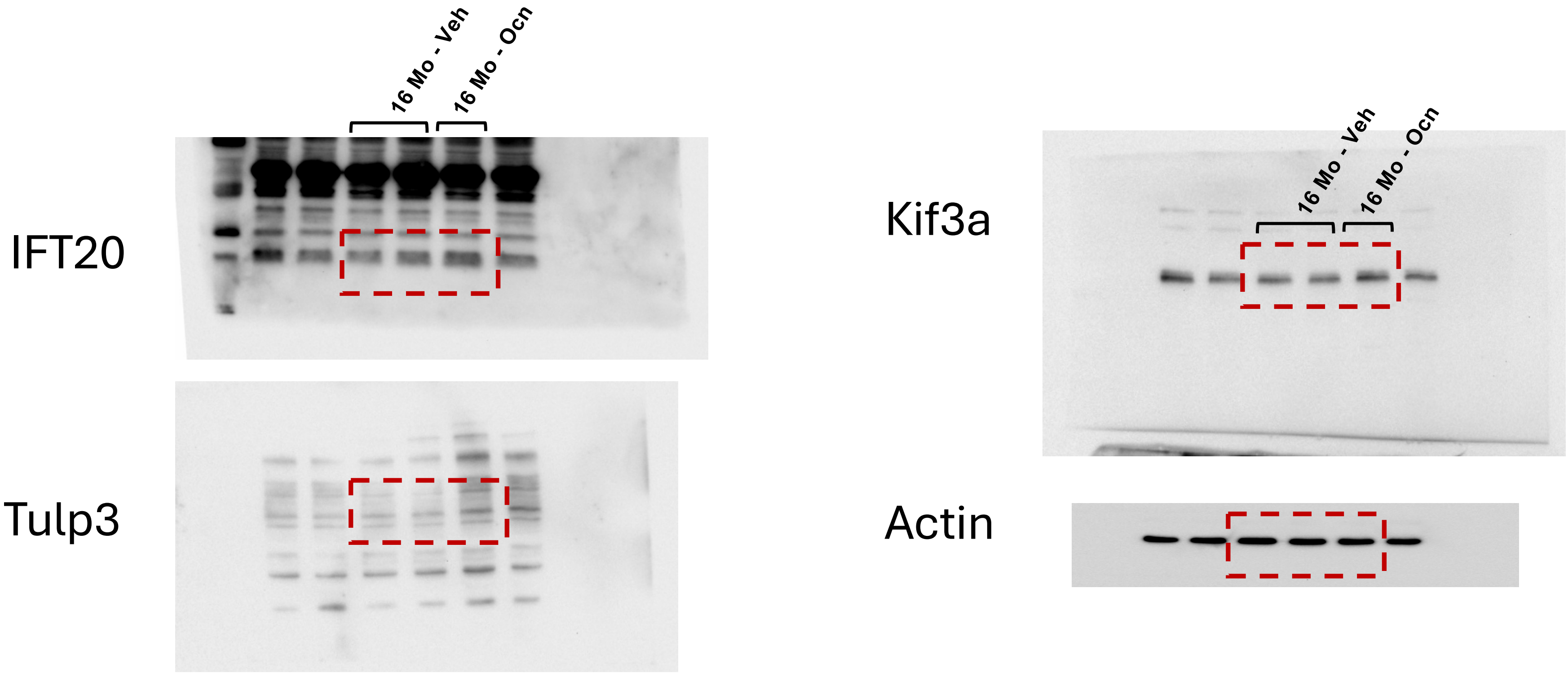

Supplement: Supplementary file 17 — Unprocessed western blots. [file 43587_2024_791_MOESM17_ESM.pdf]
